# Efficacy and mechanism of the combination of PARP and CDK4/6 inhibitors in the treatment of triple-negative breast cancer

**DOI:** 10.1186/s13046-021-01930-w

**Published:** 2021-04-08

**Authors:** Xiuzhi Zhu, Li Chen, Binhao Huang, Xiaoguang Li, Liu Yang, Xin Hu, Yizhou Jiang, Zhimin Shao, Zhonghua Wang

**Affiliations:** 1 Department of Oncology, Shanghai Medical College, Fudan University, 130 Dong-An Road, Shanghai, 200032 People’s Republic of China; 2 Key Laboratory of Breast Cancer in Shanghai, Fudan University Shanghai Cancer Center, 270 Dong-An Road, Shanghai, 200032 People’s Republic of China; 3 Department of Breast Surgery, Fudan University Shanghai Cancer Center, 270 Dong-An Road, Shanghai, 200032 People’s Republic of China; 4 Department of Gastric Surgery, Fudan University Shanghai Cancer Center, 270 Dong-An Road, Shanghai, 200032 People’s Republic of China; 5 Precision Cancer Medicine Center, Shanghai, 200032 China; 6 Institutes of Biomedical Science, Fudan University, Shanghai, 200032 China; 7 Department of Breast Surgery, Key Laboratory of Breast Cancer in Shanghai, Fudan University Shanghai Cancer Center, Shanghai, 200032 China

**Keywords:** Triple-negative breast cancer, PARP inhibitors, CDK4/6 inhibitors, Wnt signalling pathway, Resistance

## Abstract

**Background:**

PARP inhibitors (PARPi) benefit only a fraction of breast cancer patients with BRCA mutations, and their efficacy is even more limited in triple-negative breast cancer (TNBC) due to clinical primary and acquired resistance. Here, we found that the efficacy of the PARPi olaparib in TNBC can be improved by combination with the CDK4/6 inhibitor (CDK4/6i) palbociclib.

**Methods:**

We screened primary olaparib-sensitive and olaparib-resistant cell lines from existing BRCA^mut^/TNBC cell lines and generated cells with acquired olaparib resistance by gradually increasing the concentration. The effects of the PARPi olaparib and the CDK4/6i palbociclib on BRCA^mut^/TNBC cell lines were examined in both sensitive and resistant cells in vitro and in vivo. Pathway and gene alterations were assessed mechanistically and pharmacologically.

**Results:**

We demonstrated for the first time that the combination of olaparib and palbociclib has synergistic effects against BRCA^mut^/TNBC both in vitro and in vivo. In olaparib-sensitive MDA-MB-436 cells, the single agent olaparib significantly inhibited cell viability and affected cell growth due to severe DNA damage. In olaparib-resistant HCC1937 and SUM149 cells, single-agent olaparib was ineffective due to potential homologous recombination (HR) repair, and the combination of olaparib and palbociclib greatly inhibited HR during the G2 phase, increased DNA damage and inhibited tumour growth. Inadequate DNA damage caused by olaparib activated the Wnt signalling pathway and upregulated MYC. Further experiments indicated that the overexpression of β-catenin, especially its hyperphosphorylation at the Ser675 site, activated the Wnt signalling pathway and mediated olaparib resistance, which could be strongly inhibited by combined treatment with palbociclib.

**Conclusions:**

Our data provide a rationale for clinical evaluation of the therapeutic synergy of the PARPi olaparib and CDK4/6i palbociclib in BRCA^mut^/TNBCs with high Wnt signalling activation and high MYC expression that do not respond to PARPi monotherapy.

**Supplementary Information:**

The online version contains supplementary material available at 10.1186/s13046-021-01930-w.

## Background

Breast cancer (BC) is the most common malignancy and the leading cause of cancer-related death among women worldwide [1, 2]. Triple-negative breast cancer (TNBC), which accounts for approximately 15% of all BC, lacks oestrogen receptor (ER) and progesterone receptor (PR) expression and human epidermal growth factor receptor 2 (HER2) amplification [3]. Compared to other BC subtypes, TNBC exhibits inherently aggressive clinical behaviour and does not respond to endocrine therapy or anti-HER2 targeted therapy. TNBC patients typically have a poorer outcome. Chemotherapy is the primary established systemic treatment for TNBC [4]. Currently, the clinical targeted drugs for BC include poly-(ADP)-ribose polymerase (PARP) inhibitors (PARPi), CDK4/6 inhibitors (CDK4/6i), PI3K inhibitors, and AKT inhibitors, but none of these drugs alone is very effective against TNBC. There is an urgent need for the rational exploration of drug compatibility and potential targets for TNBC in the future [5, 6].

PARPi are small molecule inhibitors of the PARP family of DNA repair enzymes [7]. Tumour cells that lack BRCA1 or BRCA2 are deficient in error-free homologous recombination (HR), and DNA double-stranded breaks (DSBs) accumulated during DNA replication need to be repaired by alternative, error-prone repair pathways, which results in incorrect repair of the DNA lesions caused by PARPi and consequent cytotoxicity [8]. PARPi are a prime example of the concept of synthetic lethality in cancer treatment.

The risk of BC for BRCA1/2 mutation carriers is 50–85%, and approximately 70% of these patients have the TNBC subtype [9]; among all TNBC patients, approximately 10–20% carry BRCA1/2 gene mutations [10]. PARPis have achieved great successes in the treatment of BRCA1/2-mutated tumours, such as ovarian cancer [11] and BC [12]. In 2018, the PARPi olaparib and talazoparib were both approved by the Food and Drug Administration (FDA) for the treatment of germline BRCA-mutated (BRCA^mut^) and HER2-negative metastatic BC. Unfortunately, PARPi resistance has proven to be a major problem in the clinical treatment of BC [8], especially TNBC. In our clinical trial FUTURE, all three TNBC patients who had germline BRCA1/2 mutations were treated with PARPi and had progressed at the first evaluation [6]. Therefore, identifying who to treat, combating drug resistance and optimizing combination therapy will be the focus of research on PARPi [13]. Rational drug combinations that include PARPi may expand the patient population who may benefit from this drug class [14].

Cyclin and cyclin-dependent kinase (CDK) play an important role in the progression of the cell cycle. CDK4/6 activation regulates the transition of the cell cycle from G1 phase to S phase [15]. The CDK 4/6 inhibitor (CDK4/6i) palbociclib, which arrests cells in G1 phase, has been approved by the FDA for the treatment of ER-positive advanced BC [16–19] and is expected to provide new treatment strategies for TNBC patients, especially the luminal androgen receptor (LAR) subtype of TNBC [20].

A recent study showed that CDK4/6i prevented recovery from multiple DNA-damaging agents [21]. Another study showed that the combination of CDK4/6i and PARPi might have a potential effect in ovarian cancer [22], but the specific mechanism remains unknown. In this article, we first tested the hypothesis that the combination of the CDK4/6i palbociclib and the PARPi olaparib synergistically inhibits the growth of TNBCs, thereby providing new therapeutic opportunities for certain TNBC populations.

## Materials and methods

### Cell culture and reagents

The human BC MDA-MB-231, CAL-148, MDA-MB-453, MDA-MB-157, MDA-MB-436, and HCC1937 cell lines were obtained from Nanjing CoBioer Biosciences (CoBioer, China) in 2018. The SUM149 and MDA-MB-468 cell line were obtained from the Cell Bank of the Chinese Academy of Sciences (Shanghai, China). The human embryonic kidney HEK293T cell line was kindly provided by Prof. Guo-Hong Hu (Shanghai Institutes for Biological Sciences) in 2014. All of the above cell lines were authenticated by DNA profiling (short tandem repeat, STR), morphology, cell viability, isoenzymes, and mycoplasma assays. MDA-MB-436 cells were maintained in Leibovitz’s L15 medium (BasalMedia, L620) with 10 μg/ml insulin and 16 μg/ml glutathione, HCC1937 cells were maintained in RPMI 1640 medium (BasalMedia, L210), and SUM149 cells were maintained in DMEM/F12 (BasalMedia, L310) with 1 μg/ml hydrocortisone and 5 μg/ml insulin. The other cells were maintained in DMEM (BasalMedia, China, L110). All media were figure mented with 10% foetal bovine serum (Gibco, USA, 10270–106) and 1% penicillin-streptomycin (BasalMedia, S110B), and the cells were not passaged more than six times from collection to use. Olaparib (AZD2281, S1060), veliparib (ABT-888, S1004), palbociclib (PD-0332991, S1579), and ribociclib (LEE011, S7440) were purchased from Selleck (USA). For the administration of various inhibitors, cells were treated with 10 μM H-89 from Med Chem Express (MCE, USA, HY-15979) and 10 μM MG-132 (MCE, HY-13259) as indicated.

Acquired olaparib-resistant HCC1937 cells were generated by culturing cells in medium supplemented with increasing concentrations of olaparib for 8 months starting at 0.5 μM and reaching a final dose of 20 μM. Resistant cells were maintained in medium supplemented with 20 μM olaparib. Finally, the IC50 of HCC1937 cells with acquired resistance to olaparib was nearly three times that of the parent cells, reaching 180 μM.

### Cell viability assay and determination of drug synergy

The cells of interest (1 × 10^3^ ~ 3 × 10^3^ cells per well) were seeded into 96-well plates overnight in 100 μl of complete growth medium and then treated with the indicated drugs for the designed time in triplicate. Cell viability was tested using the cell counting kit-8 (CCK-8) assay (Dojindo Molecular Technologies, Japan, CK04) according to the manufacturer’s instructions. The combination index (CI) was used to evaluate the synergistic effect of the two drugs and was calculated by the Chou-Talalay method with CompuSyn software [23].

### Flow cytometry analysis

Flow cytometry analysis was used to assess cell apoptosis and the cell cycle. The cells of interest were treated with the designed drugs for 3 days and digested with EDTA-free trypsin. For apoptosis evaluation, the cells were collected and stained using an Annexin V-FITC/PI apoptosis kit (MultiSciences, Hangzhou, China, AP101). For cell cycle analysis, the cells were fixed using 70% precooled ethanol at 4 °C overnight, washed with PBS and stained with PI solution (MultiSciences, AP101–60-PI). The above cells were all identified and quantified by a flow cytometer (Beckman Cytomics FC 500 BD FACSCanto II) according to the manufacturer’s instructions, and the data were analysed by FlowJo v10 software.

### Migration assay

The cells used for the Transwell (Corning, USA) migration assay were pretreated with specific drugs for 3 days. Then, a total of 5 ~ 10 × 10^4^ cells were resuspended in the upper compartment of each chamber with 100 μl of serum-free medium, and the lower chamber was filled with 600 μl of medium containing 20% foetal bovine serum. The Transwell plates were incubated in a humidified environment with 95% air and 5% CO_2_ at 37 °C for 18 h (HCC1937) or 24 h (MDA-MB-436). The chambers were then washed with PBS, fixed with formaldehyde and stained with 0.5% crystal violet. The migrated cells were imaged and counted using ImageJ (National Institutes of Health, Bethesda, MD, USA).

### Senescence assay

Following the manufacturer’s protocol, a β-Galactosidase Staining Kit (Solarbio, Beijing, China, G1580) was used to detect cell senescence according to Dimri et al. [24]. Images were taken in transmitted light by a computerized imaging system consisting of a Leica charge-coupled device (CCD) DFC420 camera and a Leica DM IRE2 microscope (Leica Microsystems Imaging Solutions Ltd).

### Colony formation assay

For the colony formation assay, 500 cells were plated into 6-well or 12-well plates. After overnight incubation, the cells were treated with the designed drugs for 12–17 days, and the medium was replaced every 3 days. The colonies were fixed in formaldehyde and stained with 0.5% crystal violet. Colonies consisting of 50 or more cells were included in the count.

### Immunofluorescent staining

Immunofluorescent staining was carried out as described previously [25, 26]. Briefly, after drug treatment, the adherent cells were washed in PBS, fixed in 4% paraformaldehyde, permeabilized in 0.1% Triton X-100, and blocked in 1% bovine serum albumin (BSA) in PBS. The cells were incubated with anti-ɣH2AX (Abcam, England, ab26350, 1:250) and anti-RAD51 (Abcam, ab133534, 1:500) or anti-β-Catenin (Cell Signaling Technology, USA, D10A8 #8480, 1:100) antibodies in 1% BSA at 4 °C overnight, washed three times in PBS for 5 minutes each, and then incubated with the appropriate secondary antibody (Jackson ImmunoResearch, USA, 115–095-003/111–585-003). DNA staining was performed using Gold Antifade Mountant with DAPI (Invitrogen, USA, P36931). Leica SP5 confocal laser scanning microscopy (Leica Microsystems, Buffalo Grove, USA) was used for immunofluorescence imaging.

### HR assay

The HR assay has been described in previous research [27, 28]. The principle and steps of HR repair determination are as follows: First, transfect the DNA DSBs reporter plasmid (DR-GFP) into the cells. The DR-GFP carries the DNA sequence recognized by restriction endonuclease I-SceI. Then select cells stably expressing DR-GFP and transiently transfect another plasmid expressing I-SceI. The expressed I-SceI directly cleaves the recognized DNA sequence in DR-GFP, causing DNA DSBs. DR-GFP contains two green fluorescent protein (GFP) fragments. One of the GFP gene fragments is the full GFP gene sequence-SceGFP, but a DNA sequence recognized by I-SceI is inserted in its open reading frame; the other GFP gene fragment iGFP is located in the SceGFP downstream, without the start and end sequences of the gene. Therefore, these two GFP gene fragments actually have no biological activity. However, when the DNA DSBs are repaired by HRR, the iGFP fragment provides a repair template for HRR, so the cell will express the biologically active GFP.

In our experiment, for HR assays, a clone stably expressing pDR-GFP was generated and validated by analysing GFP-positive cells. Twenty-four hours post ISceI-GR transfection, the cells were treated with 10 μmol/L triamcinolone acetonide (TA) and cultured for another 48 h [29]. To study the effect of drugs on HR, we added a specific concentration of drugs together with TA. The proportion of GFP-positive cells was evaluated using flow cytometry, and the efficiency of HR was calculated.

### RNA-Seq analysis

Total RNA from MDA-MB-436 and HCC1937 cells treated with DMSO, 5 μM olaparib, 5 μM palbociclib, or their combination (Ola/Palb) for 24 h was extracted using TRIzol Reagent (Invitrogen). RNA quantity and quality were assessed by an Agilent 2100 Bioanalyzer (Agilent Technologies, Palo Alto, CA, USA), NanoDrop (Thermo Fisher Scientific Inc.) and 1% agarose gel. One microgram of total RNA with an RNA integrity number (RIN) value above 6.5 was used for cDNA library construction. The cDNA libraries created from the cell lines (16 samples in total, with duplicate libraries for each sample) were run on a HiSeq 2500 instrument according to the manufacturer’s instructions (Illumina, San Diego, CA, USA).

Gene set enrichment analysis (GSEA) was performed by the JAVA program using MSigDB Hallmark gene set collection. One thousand random sample permutations were carried out, and the significance threshold was set at NES absolute value> 1, NOM *p*-value< 0.05 and FDR q-value< 0.05.

### Real-time quantitative reverse transcription PCR (qRT-PCR)

Total RNA was isolated from specimens or cells using the RNeasy Plus Mini Kit (QIAGEN, Germany) following the manufacturer’s protocol. cDNA was synthesized using a PrimeScript RT Reagent Kit with gDNA Eraser (TaKaRa, Japan, RR047A). Real-time qRT-PCR was carried out using SYBR Premix Ex Taq (TaKaRa, RR420A) in triplicate on an ABI 7900HT Fast Real-Time PCR System (Applied Biosystems). All primers were synthesized by Sangon Biotech, and the primer sequences are available in Supplementary Table S1. The results were analysed with SDS v2.1 software and the 2^−ΔΔCT^ method by normalization to GAPDH levels.

### Western blot (WB) analysis

The WB protocol has been described in detail previously [30]. In short, cells were lysed in Pierce T-PER® Tissue Protein Extraction Reagent (Thermo Fisher Scientific Inc.) containing protease and phosphatase inhibitors (Bimake, USA, B14001, B15001A + B). The lysates were centrifuged at 12000 rpm for 15 min, the supernatants were collected, and the protein concentrations were determined with a Bicinchoninic (BCA) Protein Assay Kit (Solarbio, PC0020). A total of 20–30 μg of protein was separated by SDS-PAGE and transferred to PVDF membranes (Millipore, USA, IPVH00010, ISEQ00010). The primary and secondary antibodies are described in Supplementary Table S2. For the immunoprecipitation (IP) analysis, the cells were lysed in NP-40 lysis buffer (Beyotime, China, P0013F) containing protease and phosphatase inhibitors. The supernatants were incubated with 1–3 μg of primary antibodies overnight at 4 °C on a rotating platform, followed by immunoblotting analysis. ImageJ was used to quantify the immunoblotting results by measuring the protein band densities.

### Expression vectors, plasmid transfection and lentiviral infection

The CTNNB1 (NM_001904) and MYC (NM_002467) cDNAs were obtained from GeneChem (Shanghai, China) and subcloned into the Ubi-MCS-3FLAG-CBh-gcGFP-IRES-puromycin vector (GV492) to generate the Flag-gcGFP-CTNNB1 and Flag-gcGFP-MYC expression vectors, respectively. The site-directed mutations CTNNB1S675A and CTNNB1S675D were generated by PCR-based mutagenesis and verified by DNA sequencing. Human CTNNB1 short hairpin RNAs (shRNAs) in the U6-MCS-Ubiquitin-Cherry-IRES-puromycin vector (GV298) and MYC shRNAs in the U6-MCS-Ubiquitin-EGFP-IRES-puromycin vector (GV248) were purchased from GeneChem. Detailed information concerning DNA constructs and the primers used for molecular cloning is provided in Supplementary Table S1. To generate stable cell lines expressing cDNAs or shRNAs, each lentiviral expression vector was transfected into HEK293T cells with polyethyleneimine (PEI). The supernatant containing viruses was collected 48 h after transfection, filtered, and used to infect target cells in the presence of 10 μg/ml polybrene (Sigma-Aldrich, USA, H9268) prior to drug selection with 1–2 μg/ml puromycin for 1 week. Overexpression (OE) and knockdown (KD) efficiencies were validated by immunoblotting after transfection.

The lentiCas9-Blast (Addgene, USA, #52962) and lentiGuide-Puro (Addgene, #52963) vectors were provided by the Feng Zhang laboratory. The MYC knockout (KO) cell line was generated using the CRISPR/Cas9 system [31] and validated by Sanger sequencing and immunoblotting analysis. The individual sgRNA sequences are provided in Supplementary Table S3.

### Xenograft in vivo model

All animal experiments were performed in compliance with the *NIH Guide for the Care and Use of Laboratory Animal*s (http://oacu.od.nih.gov/regs/index.htm) and were approved by the Fudan Animal Ethics Committee (approval number, 201911004S). MDA-MB-436 and HCC1937 cells (8 × 10^6^ per mouse) were harvested and resuspended in 50 μl of PBS and 50 μl of Corning® Matrigel® (BD Biocoat, USA, 354248). Then, the cells were injected directly into the mammary fat pads of six-week-old female NOD-SCID mice weighing 15 to 16 g. At a room temperature of 23 °C ± 1 °C, humidity of 60% ± 5%, and environmental noise of less than 60 dB, mice were maintained in a light-dark cycle (12 h light and 12 h darkness, with lights being switched on at 7:00 a.m., light intensity of 100–150 lx at the cage level). There were 5 mice in each cage, and they were given free access to a standard rodent diet and water. Tumour volumes were monitored via calliper measurements every 3–4 days and were calculated as follows: length × width ^2^/2. When tumours reached approximately 75 mm^3^, the mice were randomly allocated into four groups, with 5 mice in each group. Four groups were administered control solvent, olaparib, palbociclib, or their combination (Ola/Palb) once a day at 9:30 p.m. Olaparib was dissolved in 4% DMSO+ 30% PEG300 + ddH_2_O for intraperitoneal administration and dosed at 50 mg/kg/day; palbociclib was dissolved in 0.9% warm saline and administered via oral gavage at 100 mg/kg/day. Euthanasia of the mice and collection of tumours were performed after the humane endpoint.

### Immunohistochemistry (IHC) analysis

Details on the IHC protocol have been described previously [32–34]. For haematoxylin and eosin (H&E) staining, slides were stained with Mayer’s haematoxylin (Sigma-Aldrich) and 0.1% sodium bicarbonate and counterstained with Eosin Y solution (Sigma-Aldrich). The primary and peroxidase (HRP)-conjugated secondary antibodies used for IHC are listed in Supplementary Table S2. The positive-staining density was measured by the computerized imaging system mentioned above.

### Statistical analysis

Quantification and statistical analysis were performed with GraphPad Prism 7.0 (GraphPad Software, Inc.), SPSS version 22.0 (SPSS, Chicago, IL) or R software version 3.5.3 utilizing the statistical tests described in the text and figure legends. The unpaired two tailed Student’s t-test was used to compare data between two groups, and correlation coefficients were calculated using the Pearson test. All *p*-values were two-sided, and p-values less than 0.05 were considered statistically significant.

## Results

### Olaparib and palbociclib synergistically inhibit the growth of BRCA^mut^/TNBCs, especially olaparib-resistant cell lines

**Fig. 1:**
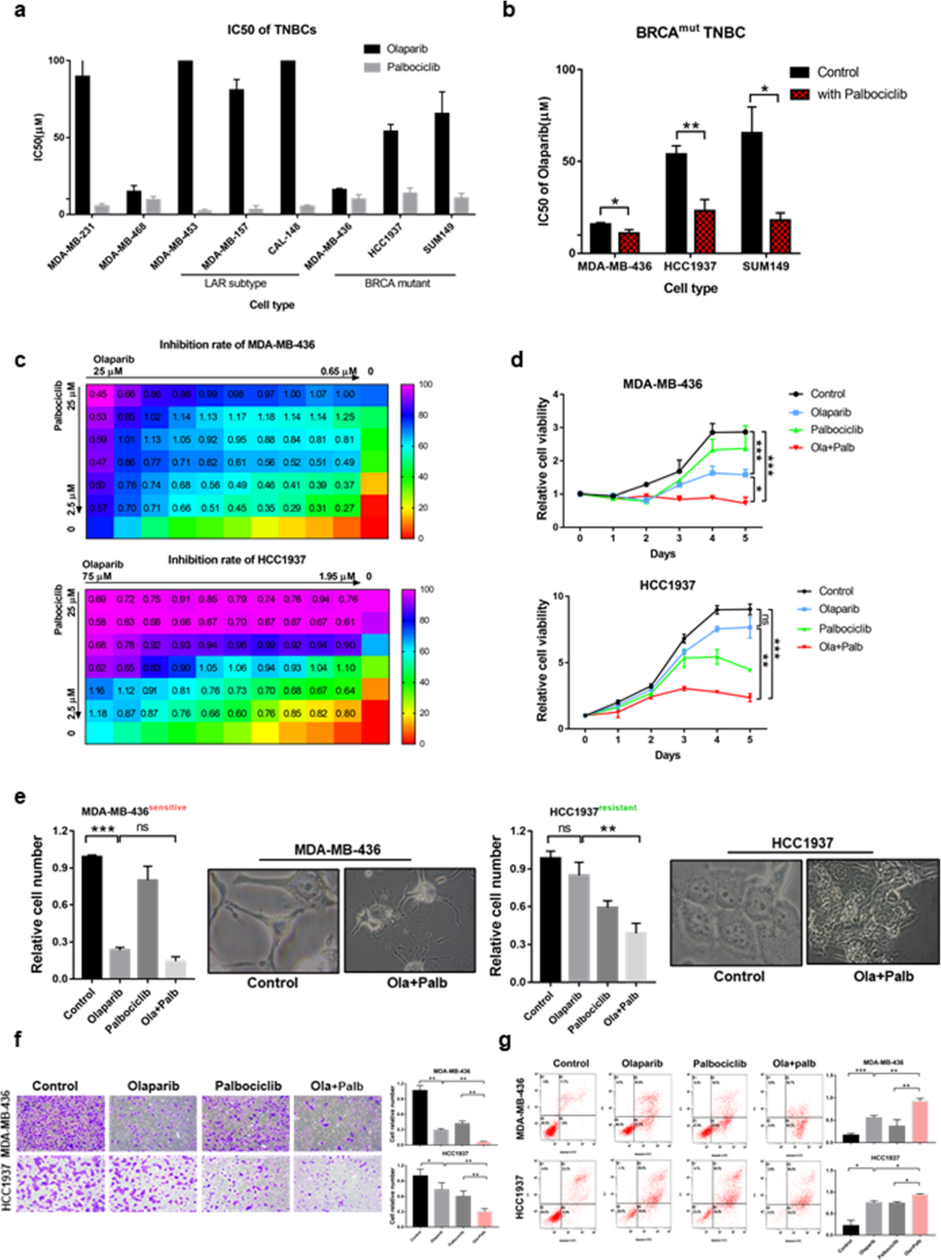
The synergistic effect of olaparib and palbociclib in BRCA^mut^/TNBCs. **a** The IC50 of olaparib and palbociclib in a panel of 8 TNBC cell lines. **b** The IC50 of olaparib was measured in BRCA^mut^ cell line with 1 μM palbociclib or not. When detecting the IC50 of olaparib in cells treated with 1 μM palbociclib, the control group used for normalization was with 1 μM palbociclib but not olaparib. **c** Inhibition rate and combination index (CI) in (*upper*) MDA-MB-436 and (*lower*) HCC1937 cells at different drug concentrations (μM). The colour represents the degree of inhibition, and the displayed value represents the CI. Olaparib was diluted 2/3 times from left to right, and the concentration of palbociclib (μM) from top to bottom was 25, 20, 15, 10, 5, 2.5, and 0. **d** Cell proliferation curve of olaparib or palbociclib alone or in combination in (*upper*) the PARPi-sensitive cells MDA-MB-436 and (*lower*) PARPi-resistant cells HCC1937. c (olaparib) = 5 μM, c (palbociclib) = 5 μM. **e** Changes in relative cell numbers and cell morphology after 3 days of single-agent or combined treatment in MDA-MB-436 and HCC1937 cells. **f** Transwell migration assay in MDA-MB-436 and HCC1937 cells pretreated with drug as indicated for 3 days. The migration times of HCC1937 and MDA-MB-436 cells were 18 h and 24 h, respectively. **g** Apoptosis evaluation after 3 days of single-agent or combined treatment in MDA-MB-436 and HCC1937 cells. c (olaparib) = 15 μM, c (palbociclib) = 15 μM. Student’s t-test; ***p < 0.001, **p < 0.01, *p < 0.05; ns, not significant. The data are presented as the means ± SEMs. Ola: Olaparib; Palb: Palbociclib

To evaluate the effects of the concomitant inhibition of PARP and CDK4/6, we assessed the response of a panel of 8 TNBC cell lines, including 3 LAR subtype cell lines and 3 BRCA^mut^ cell lines, to olaparib and palbociclib as single agents and in combination. Following a 5-day drug treatment, the half-maximal inhibitory concentration (IC50) of the two drugs was determined by the CCK-8 assay (Fig. 1a). Consistent with previous literature, the IC50 of the CDK4/6i palbociclib in the LAR subtype was lower than that in other TNBC subtypes (IC50: 9.523 ± 1.317 μM vs 3.203 ± 0.9261 μM, *p* = 0.0153). However, there was no statistically significant difference in the IC50 of the PARPi olaparib in BRCA^mut^ cells and wild-type cells (IC50: 95.26 ± 25.38 μM vs 45.04 ± 15.01 μM, *p* = 0.2077). Among BRCA^mut^ cells, there were some cell lines (HCC1937 and SUM149) resistant to olaparib, and their IC50 of olaparib was much greater than that in BRCA^mut^ MDA-MB-436 (MB436) cells, which were considered to be sensitive to olaparib. Interestingly, 1 μM palbociclib itself did not greatly affect the cell viability (the inhibition rate in BRCA^mut^ TNBCs was less than 10%), but it significantly reduced the IC50 of olaparib in BRCA^mut^ cell lines, especially those olaparib-resistant cell models (Fig. 1b).

Next, olaparib-resistant HCC1937 cells and olaparib-sensitive MB436 cells were selected for follow-up experiments. The concentration gradient design in Fig. 1c was based on the IC50 in Fig. 1a. The CompuSyn model was used to evaluate the synergistic effects of palbociclib and olaparib in the two cell lines. With increasing concentrations of olaparib and palbociclib, the growth inhibition of MB436 and HCC1937 cells became stronger, and the CIs were 0.72 ± 0.25 and 0.80 ± 0.13, respectively, suggesting the potential synergistic effect of the inhibitors (Fig. 1c and S1a).

Next, we chose a drug combination with olaparib = 5 μM and palbciclib = 5 μM, which greatly inhibit cell growth at a relatively small concentration in olaparib-resistant cells, while the respective single drug did not (inhibition rate: < 15% for drug alone but > 50% for combination, Supplementary Table S4). In olaparib-sensitive MB436 cells, the single agent olaparib significantly inhibited cell viability and affected cell growth, while in olaparib-resistant HCC1937 and SUM149cells, only the combination treatment had a significant inhibitory effect (Fig. 1d and e, Fig. S1b and S1c). Under the combined treatment, reduced synapses between the cells and nuclear shrinkage were observed. In addition, olaparib combined with palbociclib inhibited cell migration (Fig. 1f and Fig. S1d) and promoted BRCA^mut^/TNBC apoptosis (Fig. 1g and Fig. S1e) and cell senescence (Fig. S1f) more effectively than either agent alone in BRCA^mut^/TNBC cells. To exclude the special effects of these two drugs, we tested the cell viability effects of two other drugs, the CDK4/6i ribociclib and the PARPi veliparib, in MB436 and SUM149 cells, and consistent experimental results were obtained (Fig. S1g and S1h). We found that CDK4/6i resensitized olaparib-resistant BRCA^mut^ cells to olaparib.

### Palbociclib and olaparib are synthetically lethal by inducing HR repair deficiency

**Fig. 2:**
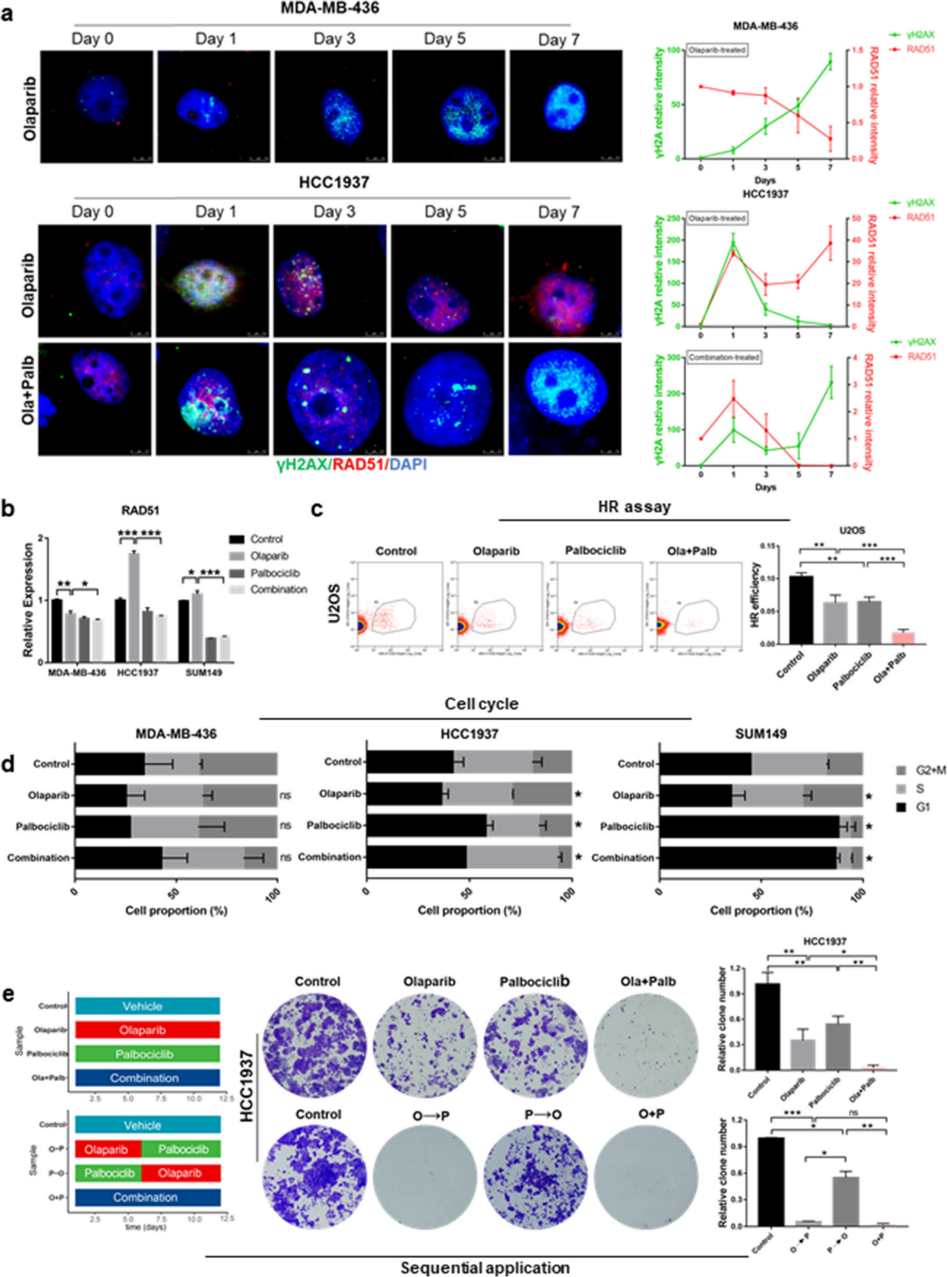
The combination of olaparib and palbociclib results in irreversible lethal DNA damage and redistribution of the cell cycle. **a** (*Left)* Representative images at different treatment time points of BRCA^mut^/TNBC cells stained with DAPI, γH2AX and RAD51. (*Up*) MDA-MD-436 cells treated with olaparib alone. (*Down*) HCC1937 cells treated with olaparib alone or olaparib plus palbociclib. (*Right)* The signal intensity of γH2AX and RAD51 in cells changing over time. Scale bar, 7.5 μm. **b** Quantitative reverse transcription PCR analysis of RAD51 mRNA expression in BRCA^mut^/TNBC cells treated with drugs as indicated in (**a**) for 72 h. **c** Efficiency of homologous recombination (HR) in U2OS cells treated with vehicle, 20 μM olaparib, 5 μM palbociclib or their combination for 72 h; *n* = 3 independent assays. **d** The percentage of cells in different phases of the cell cycle was analysed by flow cytometry. The treatment of cells was as described in (**a**). The *p*-value here is the t-test of the G2 + M phase of each group relative to the control group. **e** The effect of different sequential application was tested in colony formation assays. The effect of 6-day olaparib followed by 6-day palbociclib (O-P) and its reverse (P-O) effect were tested in HCC1937 cells. (*Left*) Pattern diagram of sequential administration. (*Middle*) Representative images of colony formation assays. (*Right*) Quantification of colony formation. Student’s t-test; ***p < 0.001, **p < 0.01, *p < 0.05; ns, not significant. The data are presented as the means ± SEMs. Con: Control; Ola: Olaparib; Palb: Palbociclib

The synergistic activity of PARPi and CDK4/6i in BRCA^mut^/TNBC cells prompted us to examine whether they induced synthetic lethality through DNA damage repair or even HR repair mechanisms. Immunofluorescence staining analysis revealed that in olaparib-sensitive MB436 cells, treatment with olaparib alone showed a significant increase in the intensity of γH2AX nuclear foci (a surrogate marker of DNA DSBs), while RAD51 foci (a marker of the competency of HR repair) were hardly observed (Fig. 2a). In contrast, there was a significant increase in the intensity of RAD51 foci but no significant changes in the intensity of γH2AX foci after treatment with olaparib alone in olaparib-resistant HCC1937 and SUM149 cells, indicating the potential HR repair ability of these cells and the protection of DNA from serious damage (Fig. S2). Although the combination of olaparib and palbociclib in HCC1937 cells caused a short-term increase in RAD51 nuclear foci, the staining decreased rapidly after 1 day of treatment and was maintained over time, while γH2AX foci cumulatively increased over time, indicative of persistent and severe DNA damage (Fig. 2a). The real-time qRT-PCR results also verified that in the olaparib-resistant model (HCC1937 and SUM149 cells), RAD51 increased under the single-agent treatment but decreased under the combined treatment; in the olaparib-sensitive model (MB436 cells), olaparib alone could maintain RAD51 at a low level (Fig. 2b) and cause DNA damage.

To confirm the effect of the palbociclib and olaparib combination on DNA repair, we next directly measured DNA HR repair by using specific fluorescent reporter assays [28, 35]. The proportion of GFP-positive cells measured the efficiency of HR. As shown in Fig. 2c, compared with the vehicle, olaparib and palbociclib alone could each reduce the level of HR repair, but HR repair was impaired more significantly in the combination treatment. These results indicated that palbociclib inhibition may induce deficiency in the HR repair of DSBs, which is a potential mechanism underlying the synthetic lethality of olaparib.

### Palbociclib results in defective G2 cell cycle-dependent DNA repair after chromosomal damage caused by olaparib

Since palbociclib and olaparib act in different phases of the cell cycle, we next analysed the effect of their combination on cell cycle progression by DNA content measurements. In olaparib-sensitive and olaparib-resistant cells, the drugs had different effects on cell cycle progression (Fig. 2d). Cell cycle analysis following olaparib exposure showed an extended G2 phase and demonstrated the absence of G1 arrest (*p* < 0.05) after palbociclib exposure in HCC1937 and SUM149 cells, which was not observed in olaparib-sensitive MB436 cells. Moreover, compared to the extended G2 phase in response to olaparib alone, the G2 phase was greatly reduced following the combined treatment. These changes in cell cycle progression might be partly due to G1 arrest of the cell cycle caused by CDK4/6i. Cells with defective p53 and BRCA expression relied on the G2 checkpoint of the cell cycle for DNA repair and triggered G2 cell-cycle arrest [36, 37]. We speculated that the reduction in some cell cycle-dependent DNA damage repair, such as in G2 phase, may cause irreversible lethal DNA damage under the combination of palbociclib and olaparib.

Since concomitant treatment with CDK4/6i and PARPi might affect the efficacy of the combined treatment, we evaluated the effect of the sequential administration of these drugs. We found that the combination treatment significantly inhibited colony formation in HCC1937 cells (Fig. 2e), whereas a 6-day pretreatment with palbociclib (P-O, Fig. 2e and S3a) reduced the antiproliferative effect of olaparib. We observed a significant decrease in colony formation in the two resistant cell lines after a 6-day pretreatment with olaparib before the continuous administration of palbociclib (O-P). Similar data were obtained with ribociclib and veliparib (Fig. S3b) in the olaparib-resistant cell lines. In MDA-MB-436 cells, 6-day pretreatment with palbociclib (P-O, Fig. S3c) also reduced the antiproliferative effect of Olaparib. Regarding the relationship between cell cycle arrest and DNA damage, we speculated that palbociclib interferes with the cytotoxic effect of DNA-damaging agents, as palbociclib arrests cells in G1, thereby protecting the cells from damage during DNA replication or mitosis, which was consistent with some previous studies [21, 38]. Under the combined treatment, the antitumour effect of palbociclib (inhibiting DNA repair and increasing DNA damage) may partly depend on the pretreatment effect of olaparib.

### Combined use of olaparib and palbociclib inhibits MYC expression through the Wnt pathway

**Fig. 3:**
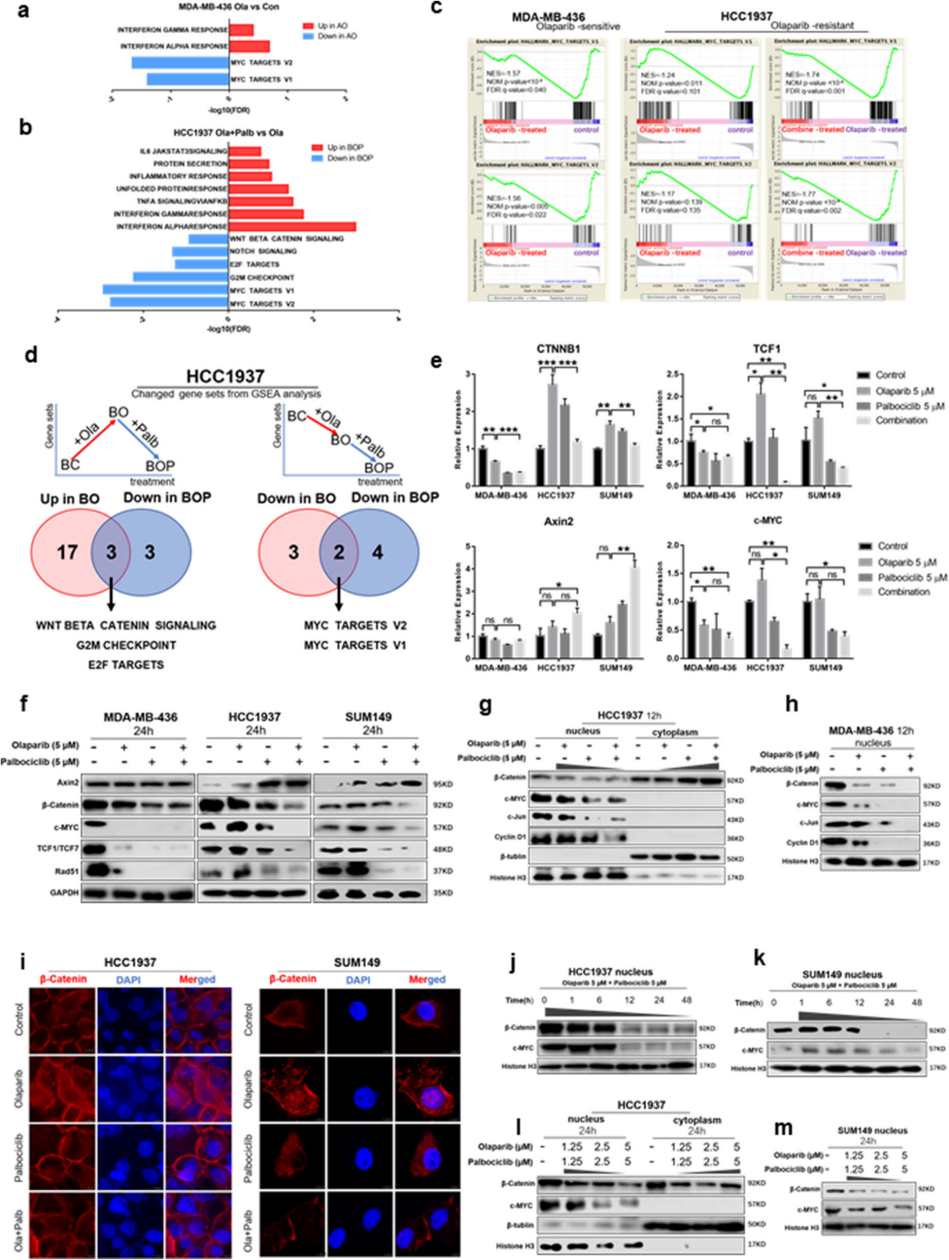
The combined use of olaparib and palbociclib inhibits MYC expression through the Wnt pathway. **a** GSEA results in olaparib-treated PARPi-sensitive MDA-MB-436 cells (AO) compared to vehicle-treated cells (AC). **b** GSEA results in combination-treated olaparib-resistant HCC1937 cells (BOP) compared to olaparib-treated cells (BO). **c** GSEA of MYC gene signatures in MDA-MB-436 (left panels) and HCC1937 (right panels) cells with different treatments. **d** (*Left)* The overlap of hallmark gene sets in GSEA, which were downregulated in combination-treated cells (BOP) but upregulated in olaparib-treated resistant HCC1937 cells (BO). (*Right)* The overlap of hallmark gene sets in GSEA that were downregulated in both BO and BOP. **e** Quantitative reverse transcription PCR analysis of CTNNB1, TCF1, Axin2 and MYC in MDA-MB-436, HCC1937 and SUM149 BRCA^mut^/TNBC cell lines treated with drugs as indicated for 24 h. Mean ± S.D. for three independent experiments. **f** WB analysis showed the total levels of β-catenin, TCF1, Axin2, c-myc and Rad51 in three BRCA^mut^/TNBC cell lines treated with drugs as indicated for 24 h. **g** WB analysis showed the nucleoplasmic distribution of β-catenin and the change in the Wnt pathway (c-myc, c-Jun and Rad51) in HCC1937 upon treatment with drugs as indicated for 12 h. **h** WB analysis showed the change in the Wnt pathway (c-myc, c-Jun and Rad51) in MDA-MB-436 upon treatment with drugs as indicated for 12 h. **i** Immunofluorescence analysis of the nucleoplasmic distribution of β-catenin in olaparib-resistant HCC1937 and SUM149 cells treated with drugs as indicated for 12 h. Scale bar, 7.5 μm. **j** Changes in nuclear β-catenin and c-myc protein levels in HCC1937 over time under the indicated treatment. **k** Changes in nuclear β-catenin and c-myc protein levels in SUM149 over time under the indicated treatment. **l** Changes in the nucleoplasmic distribution of β-catenin and c-myc in HCC1937 under different drug concentrations for 24 h. **m** Changes in the nuclear distribution of β-catenin and c-myc in SUM149 under different drug concentrations for 24 h. Student’s t-test; ***p < 0.001, **p < 0.01, *p < 0.05; ns, not significant. The data are presented as the means ± SEMs. Con: Control; Ola: Olaparib; Palb: Palbociclib

We next explored in depth the molecular mechanism of the combination of olaparib and palbociclib. RNA sequencing analysis was applied to olaparib-sensitive MB436 cells and olaparib-resistant HCC1937 cells treated with vehicle, olaparib, palbociclib or a combination of the two for 24 h due to the significantly heightened expression of c-PARP and downregulation of pRB (S807/811) in 24 h (Fig. S4a). GSEA revealed that MYC target gene sets were significantly downregulated, while immune-related pathways (alpha/gamma interferon response) were significantly upregulated in MB436 cells treated with olaparib (Fig. 3a and c). In olaparib-resistant BRCA^mut^ HCC1937 cells, many MYC target genes were strongly downregulated only upon combined drug treatments, but the changes were not significant with olaparib alone (NES = -1.17, NOM *p*-value = 0.139, FDR q-value = 0.135) (Fig. 3b and c). In addition, in HCC1937 cells, DNA replication and DNA damage repair gene sets (mitotic spindle and UV response DN) were upregulated after single-agent olaparib treatment (Fig. S4b). More interestingly, the combined treatment downregulated the gene sets that were upregulated by the single agent olaparib in olaparib-resistant cell lines, such as the Wnt β-catenin signalling (Fig. S4c), G2/M checkpoint and E2F target genes (Fig. 3d). These pathways might play an important role in olaparib resistance and to some extent explain the mechanism by which palbociclib resensitizes olaparib-resistant cells.

To clarify the relationship between the Wnt pathway and MYC, we then selected β-catenin (the core molecule in the Wnt pathway) and c-myc for WB analysis in HCC1937 and SUM149 cell lines. We found that knockdown or overexpression of c-myc did not affect the expression of β-catenin. However, the expression of c-myc decreased in CTNNB1-knockdown cells and increased with β-catenin overexpression. Therefore, we confirmed that the Wnt pathway acted as upstream to regulate the expression of c-myc (Fig. S4d-4g), which was consistent with some previous studies [39, 40]. Subsequent qRT-PCR and WB analyses confirmed that treatment with olaparib alone for 24 h in olaparib-sensitive MB436 cells resulted in significant downregulation of Wnt β-catenin signalling; in olaparib-resistant HCC1937 and SUM149 cells, treatment with olaparib alone resulted in marked upregulation of Wnt β-catenin signalling, which were downregulated (decrease of β-catenin, TCF1, c-myc and increase of Axin2) by the combined treatment with olaparib and palbociclib (Figs. 2b, 3e and f).

The entry of β-catenin into the nucleus plays a central role in the activation of the classical Wnt pathway. In-depth WB analysis revealed that the nucleoplasmic distribution of β-catenin changed before an obvious change was found in the total level of catenin protein (Fig. S4h and Fig. 3g-i). The 12 h combined treatment prevented β-catenin from entering the nucleus in HCC1937 cells (Fig. 3g) and reduced the expression of MYC, although the total amount of β-catenin was not significantly reduced (Fig. S4h). Moreover, the expression of MYC was positively correlated with the level of β-catenin in the nucleus, and the effects were both time and dose dependent in BRCA^mut^/TNBC (Fig. 3j-m and Fig. S4i).

Previous studies have shown that DNA damage can activate the Wnt pathway. We therefore assumed that DNA damage caused by olaparib activated the Wnt signalling pathway and upregulated MYC, leading to resistance to olaparib. Overall, the combined use of olaparib and palbociclib inhibits MYC expression through the Wnt pathway.

### Ser675 phosphorylation of β-catenin in the Wnt signalling pathway mediates resistance to olaparib

**Fig. 4:**
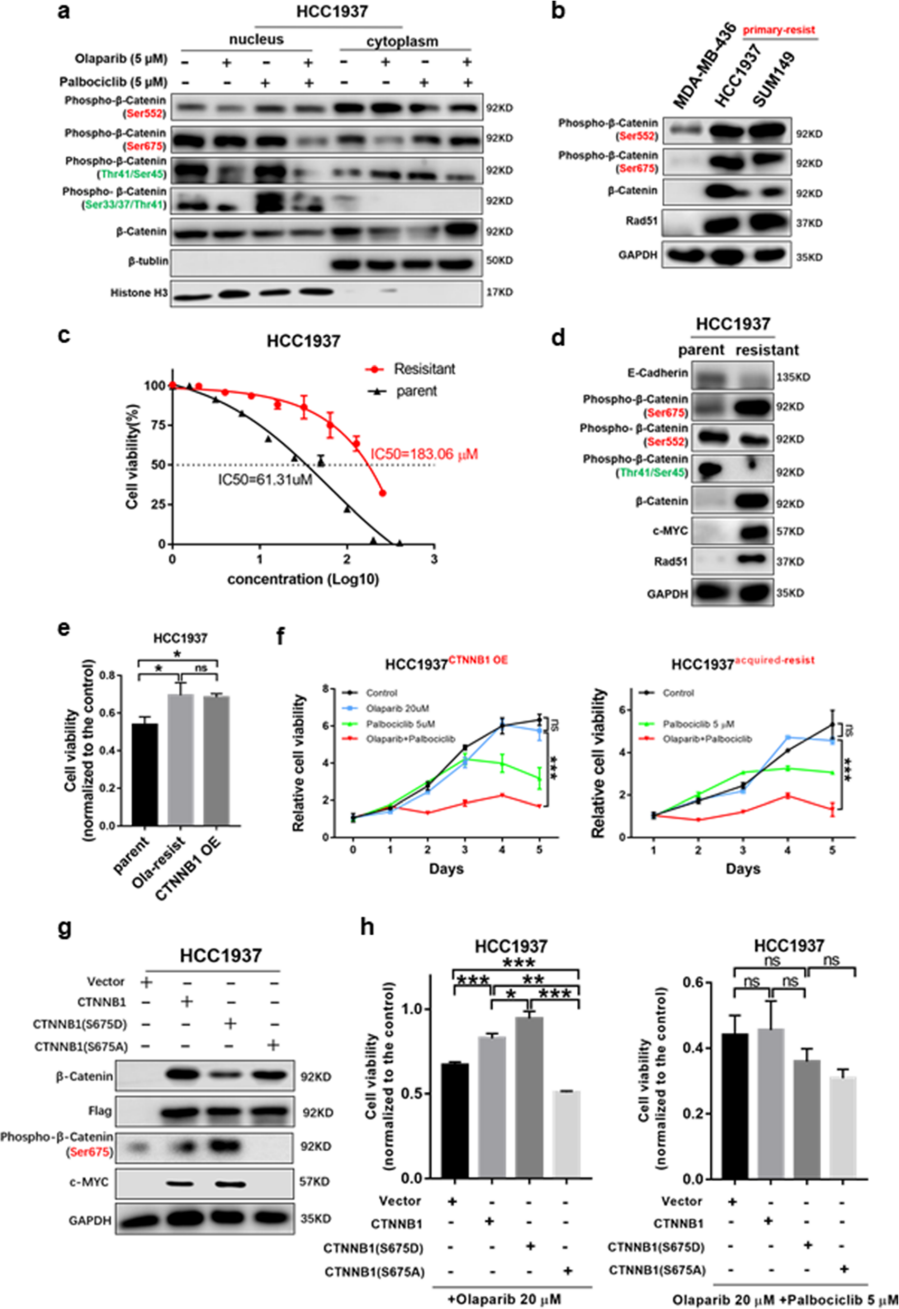
Ser675 phosphorylation of β-catenin in the Wnt pathway mediates resistance to olaparib but can be inhibited by palbociclib. **a** The phosphorylation level of specific sites of β-catenin in cells treated with drugs as indicated for 12 h. The phosphorylation sites marked in green negatively regulate the Wnt pathway, while those marked in red promote nuclear translocation, thereby positively regulating the Wnt pathway. **b** Western blot (WB) analysis showing the levels of β-catenin, phosphorylated β-catenin (pβ-cateninSer552 and pβ-cateninSer675) and RAD51 (a homologous recombination repair marker) in MDA-MB-436, HCC1937 and SUM149 cells. **c** CCK-8 analysis of parental HCC1937 cells and cells with acquired resistance to olaparib at different concentrations of olaparib. **d** Expression difference in Wnt signalling pathway-related proteins and Rad51 between parental HCC1937 and acquired olaparib-resistant cells assessed by WB analysis. **e** The viability of parental HCC1937 cells, HCC1937 cells with acquired olaparib resistance and CTNNB1 OE HCC1937 cells treated with 20 μM olaparib for 5 days. **f** Growth curves of HCC1937 and CTNNB1 OE HCC1937 cells with acquired olaparib resistance under the indicated treatments. **g** WB analysis of β-catenin, pβ-cateninSer675 and c-myc protein levels in CTNNB1 KD HCC1937 cells with or without β-catenin mutant overexpression. **h** (*Left*) The viability of the control and HCC1937 cells expressing β-catenin^WT^, mutant β-catenin^S675D^ or β-catenin^S675A^ under the indicated treatments. (*Right*) The four cell types under combined treatment for 5 days. Student’s t-test; ***p < 0.001, **p < 0.01, *p < 0.05; ns, not significant. The data are presented as the means ± SEMs. Con: Control; Ola: Olaparib; Palb: Palbociclib

The phosphorylation of different sites of β-catenin affects its nuclear accumulation, protein stability and signalling activity [41–43]. Phosphorylation at Ser33, Ser37, Thr41 or Ser45 promotes β-catenin degradation and inhibits the classical Wnt pathway, while phosphorylation at Ser552 or Ser675 stabilizes β-catenin and promotes its nuclear translocation, which positively regulates the Wnt signalling pathway. We next tested the phosphorylation levels of different sites of β-catenin under different drug treatments for 12 h. We were surprised to find that the combination treatment of olaparib and palbociclib greatly reduced phosphorylation at Ser675 in the nucleus (Fig. 4a and Fig. S5a). Compared to those in the olaparib-sensitive BRCA^mut^ TNBC MB436 cell line, Ser552 and Ser675 of β-catenin in the olaparib-resistant HCC1937 and SUM149 cells both showed higher phosphorylation, and Rad51 and β-catenin were highly expressed (Fig. 4b). In contrast, the phosphorylation sites (Ser33, Ser37, Thr41 and Ser45) that negatively regulated the Wnt signalling pathway were highly phosphorylated in MB436 cells (Fig. S5b).

Cells with acquired olaparib resistance (HCC1937) were generated by treatment with olaparib in a stepwise dose-escalating fashion for 8 months and maintained in medium supplemented with 20 μM olaparib. The CCK-8 assay verified that the IC50 of olaparib in the HCC1937 cells with acquired olaparib resistance was nearly three times that of the parent cells (Fig. 4c). Compared to the parental cell lines, HCC1937 cells with acquired resistance expressed high levels of β-catenin, c-myc and Rad51, and the Ser675 site of β-catenin was highly phosphorylated, which was positively correlated with Wnt signalling pathway activation (Fig. 4d).

Next, 20 μM olaparib was selected to treat resistant cells. 20 μM olaparib inhibited the growth of parent HCC1937 cells to a certain extent, but did not affect the growth of acquired-resistant HCC1937 cells (Fig. 4e and Fig. S6), which could be greatly inhibited under the combination treatment of 20 μM olaparib and 5 μM palbciclib (Fig. 4f). Notably, CTNNB1 overexpression in parent cells, confirmed by WB analysis (Fig. S4f), also resulted in drug resistance to olaparib (Fig. 4f).

We next generated two β-catenin mutants, S675D (Ser-to-Asp) and S675A (Ser-to-Ala), in the C-terminal domain in HCC1937 cells (Fig. 4g). Mutant β-catenin^S675D^ was an activating mutation that maintained continuous phosphorylation at site 675, while mutant β-catenin^S675A^ was an inhibitory mutation. Cells with β-catenin^WT^ or mutant β-catenin^S675D^ both showed resistance to high concentrations of olaparib, while cells with mutant β-catenin^S675A^ were much more sensitive to olaparib (Fig. 4h). Therefore, we hypothesized that overexpression of β-catenin, especially its hyperphosphorylation at the Ser675 site, activated the Wnt signalling pathway, thereby mediating olaparib resistance.

### Palbociclib can strongly inhibit the viability of olaparib-resistant cells

In both HCC1937 cells with acquired resistance to olaparib and cells with high expression of β-catenin^WT^ or mutant β-catenin^S675D^, continuous activation of the Wnt signalling pathway and high expression of MYC made high concentrations of olaparib ineffective. Interestingly, a small dose of palbociclib greatly inhibited the viability of these cells (Fig. 4f and h). In addition, the combined treatment had a stronger inhibitory effect in cells with β-catenin^WT^ or mutant β-catenin^S675D^ than in the control cell line (Fig. 4h) and made no significant difference in the viability between the cells with mutant β-catenin^S675D^ and β-catenin^S675A^.

### Olaparib and palbociclib synergistically inhibited tumour growth in vivo

**Fig. 5:**
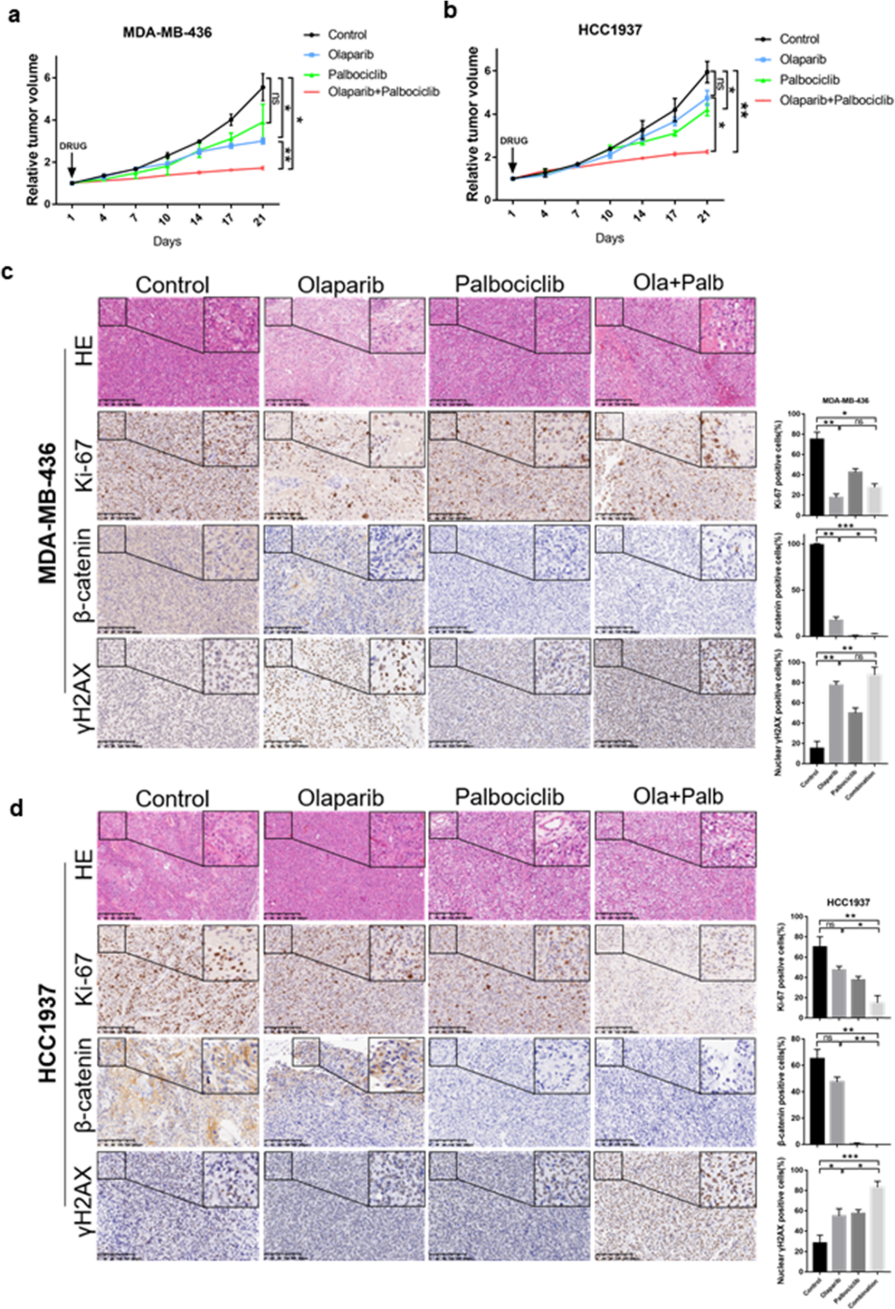
The combined treatment of olaparib and palbociclib is effective in vivo. **a** and **b** Tumour growth curves of (A) MDA-MB-436 and (B) HCC1937 xenografted NOD-SCID mice treated with olaparib (50 mg/kg/day) and palbociclib (100 mg/kg/day), either alone or in combination, for 21 days. The arrow indicates the start of treatment. **c** and **d** Representative images of immunohistochemical staining as indicated in the xenografted tumours (*n* ≥ 3 per treatment group) treated with olaparib and palbociclib either as single agents or in combination for 21 days. Scale bar, 200 μm. The small image inside is magnified 4 times relative to the large image. Student’s t-test; ***p < 0.001, **p < 0.01, *p < 0.05; ns, not significant. The data are presented as the means ± SEMs. Con: Control; Ola: Olaparib; Palb: Palbociclib

We next evaluated the efficacy of the combination of olaparib and palbociclib in a xenograft mouse model of MB436 and HCC1937 cells. We collected the tumour on the 21st day of medication due to the heavy tumour burden in the control group. Consistent with the in vitro experiments, single-agent olaparib significantly inhibited the growth of the MB436 tumour model; however, the single agent had limited activity in the HCC1937 tumour model (Fig. 5a and b). The tumour growth in both models was significantly slowed under the combination treatment of olaparib and palbociclib. In addition, we analysed the weight of the mice in each group and found no significant difference (Fig. S7). In our experiments, no obvious side effects were observed in mice, including vomiting and diarrhoea. This might suggest that, to a certain extent, the dosage and combination of olaparib and palbociclib were tolerable to these mice.

As determined by histological analysis, the combination treatment resulted in a substantial decrease in Ki-67 (a proliferation marker) and β-catenin but a significant increase in the formation of γH2AX nuclear foci (Fig. 5c and d). Decreased proliferation and increased DNA damage may, at least in part, explain the observed response to the combined treatment. Consistent with the conclusions drawn in vitro, palbociclib alone or in combination with olaparib sufficiently suppressed β-catenin expression and inhibited the Wnt signalling pathway.

## Discussion

The development of PARPi resistance in BRCA^mut^ cancers is a pressing clinical problem. Currently, resistance to PARPi can be classified into four main mechanisms: altered drug availability, affected (de) PARylation enzymes, restored HR, and restored replication fork stability [44]. To overcome resistance to PARPi and enhance their efficacy, an increasing number of studies are currently exploring treatment strategies that can be combined with PARPi, including oncolytic herpes simplex viruses (oHSVs) [45, 46], ionizing radiation [47], CDK inhibitors (CDK12i [48], CDK1/2i [49], CDK18i [50], etc.), immunotherapy [51, 52], epigenetic drugs (HDAC inhibitor [53] and DNMT inhibitor [54, 55]).

**Fig. 6:**
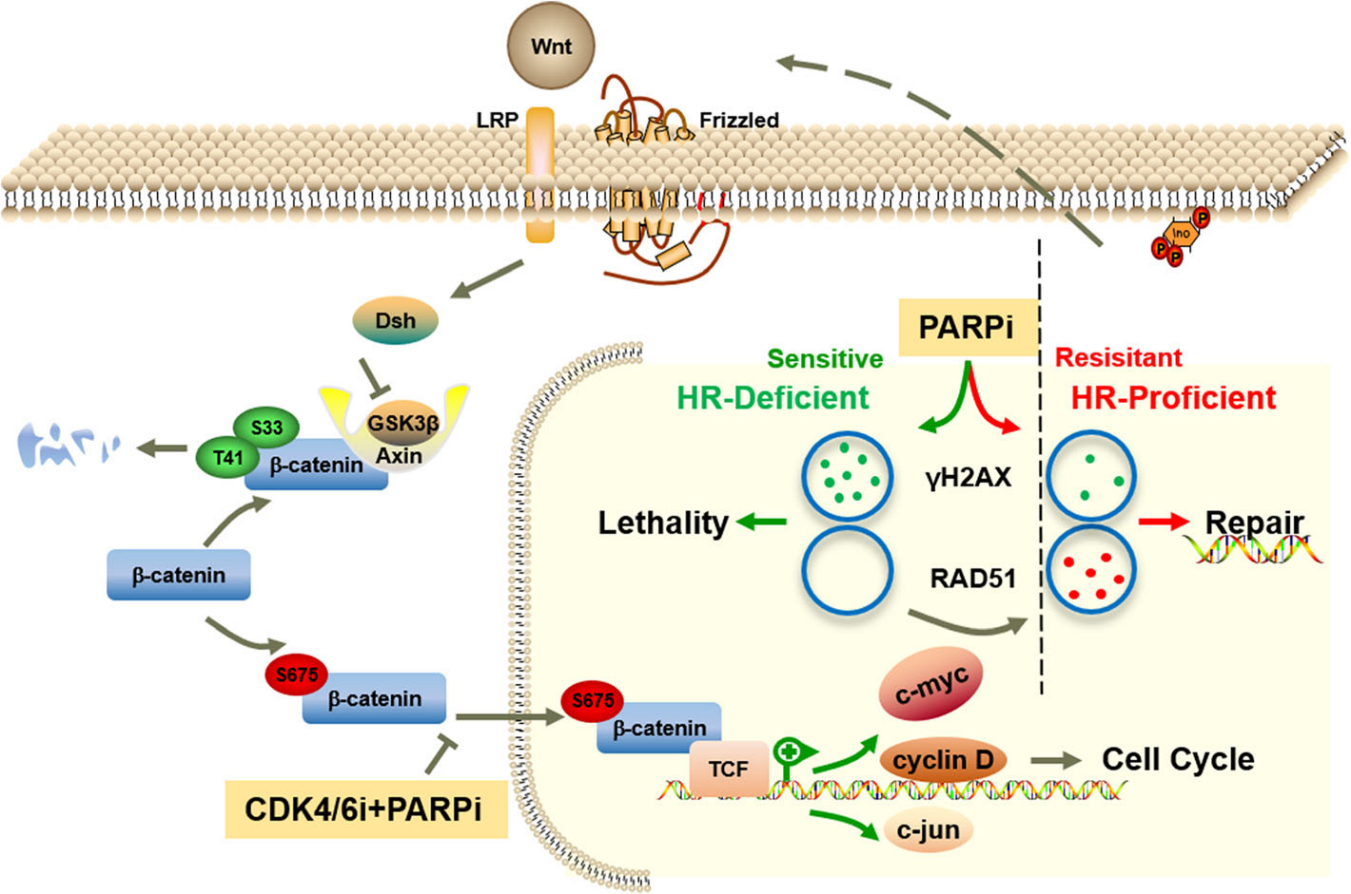
The pattern diagram of the combination of PARP and CDK4/6 inhibitors in the treatment of triple-negative breast cancer

Our study is the first to test the hypothesis that the combination of olaparib and palbociclib synergistically inhibits the growth of BRCA^mut^ TNBCs, thereby providing new treatment opportunities for certain TNBCs, especially those resistant to olaparib (Fig. 6). We selected one olaparib-sensitive (MB436) and two relatively olaparib-resistant BRCA^mut^/TNBC cell lines (HCC1937 and SUM149) out of 8 TNBC cell lines based on their response to olaparib treatment. We hoped to use the combination of drugs to greatly inhibit cell growth at a relatively small concentration in olaparib-resistant cells, while the single drug could not. In the olaparib-sensitive cells, the single agent olaparib significantly inhibited cell viability and affected cell growth in vitro and in vivo due to severe DNA damage. In the olaparib-resistant cells, potential HR repair ability and protection of DNA from serious damage were observed after treatment with the single agent olaparib, making it ineffective. Only the combination with palbociclib could greatly inhibit HR and strongly suppress tumour growth.

In addition to the changes in HR and DNA damage that we found, the cell cycle distribution changed with olaparib or palbociclib administration. Compared to the extended G2 phase in response to treatment with olaparib alone in olaparib-resistant BRCA^mut^ /TNBCs, the G2 phase and G2/M checkpoint-related gene sets were greatly reduced following the combined treatment. A previous study showed that BRCA1 loss resulted in an accumulation of G1 DNA damage [56]. In the S and G2 phases, DNA replication occurs, sister chromatids are present, and cell DNA damage can be repaired by HR [44]. We speculate that the G1 arrest of the cell cycle caused by palbociclib increases the DNA damage in the G1 phase caused by olaparib and BRCA1/2 mutations. In addition, G1 phase arrest indirectly reduces the G2 phase and G2-dependent HR. Upon sequential administration of the two drugs, we found that PARPi followed by CDK4/6i had a better inhibitory effect. The antitumour effect of CDK4/6i in the combination treatment may depend partly on the pretreatment effect of PARPi, which causes DNA damage and helps PARPi increase DNA damage.

When we were exploring the molecular mechanism of the combination treatment in depth, MYC and the Wnt signalling pathway attracted our attention and were observed to mediate resistance to olaparib mainly for the following reasons. First, the Wnt pathway was upregulated under olaparib treatment alone and downregulated under the combination treatment in olaparib-resistant cells. This may be consistent with the view that inadequate DNA damage can activate the Wnt signalling pathway [57]. Second, in olaparib-resistant cells, many MYC targets were strongly downregulated only upon combined drug treatment, while in olaparib-sensitive cells, olaparib alone was sufficient. Third, the Wnt pathway acts upstream to regulate the expression of MYC. The role of MYC in DNA damage response and genomic instability has been the subject of debate and controversy [58]. Previous studies have reported that dysregulation of MYC could induce DNA damage and genomic instability [59, 60]. We predicted that inadequate DNA damage caused by olaparib activated the Wnt signalling pathway and upregulated MYC, leading to resistance to PARPi. In addition, we found activation of the Wnt signalling pathway in cells with acquired olaparib resistance. In follow-up studies, we found that any activation of the Wnt signalling pathway, such as the overexpression of β-catenin (the core molecule in the Wnt pathway) or its hyperphosphorylation at the Ser675 site (promoting its nuclear translocation, thereby positively regulating Wnt), could mediate resistance to the PARPi olaparib but be inhibited by the combination with CDK4/6i palbociclib. Our studies could pave the way for novel treatment options to target primary and acquired olaparib resistance. How does a small dose of palbociclib act as a sensitizer for PARPi? This is a quite surprising and interesting discovery. At present, there were few reports on the mechanism of the synergy of olaparib and palbociclib. A recent study by Jingyan Yi et al. revealed that palbociclib could induce downregulation of MYC and its target genes involved in HR repair in ovarian cancer cells [22]. Another study showed that Palbociclib treatment led to the downregulation of the DNA repair pathway and the upregulation of IL-6/STAT3 pathway, and the combination using STAT3, PARP andCDK4/6 inhibitors could target these pathways and greatly inhibit tumour growth. Whether there are more interactions between olaparib and palbociclib at the regulation of cell cycle and DNA damage repair requires further in-depth research in the future.

In addition, although the weight of mice in each group showed little difference in our study, the toxicity of the combination of olaparib and palbociclib is worthy of further exploration. Previous safety analysis showed that in olaparib treatment, nausea, anaemia, vomiting, leukopenia and fatigue were the most common side effects. Among them, anaemia was the most common grade ≥ 3 adverse event [61]. For palbociclib treatment, neutropenia, leukopenia, anaemia and fatigue occurred at a higher frequency [62]. The above reminds us that we should pay more attention to the patient’s complete blood count, especially haemoglobin and leukocyte, when the two drugs are used in combination. Clinical trials are still needed to verify the side effects and safe dosage.

Our research also has some limitations. First, we did not explore the role of human immunity in drug resistance and resensitization. Second, 21-day treatment in vivo was not adequate to evaluate the efficacy against overall tumour progression, and longer-term in vivo research is needed. In addition, we did not study a clinical cohort to verify our hypothesis, and the existing database lacks medication information and cannot be used for verification.

## Conclusions

To a certain extent, our data provide a rationale for clinical evaluation of the therapeutic synergy of olaparib and palbociclib in BRCA^mut^/TNBCs that show high Wnt signalling pathway activation and high MYC expression and do not respond to PARPi monotherapies.

## Supplementary Information

**Additional file 1: Table S1.** Primers used in RT-PCR. **Table S2.** Antibodies used in this study. **Table S3.** sgRNA sequence of genes. **Table S4.** Inhibition rate and combination index of HCC1937 and MDA-MB-436 with single-agent or combined treatment.

**Additional file 2: Figure S1.** Synergistic response to combined PARPi and CDK4/6i treatment in BRCAmut/TNBCs. **Figure S2.** Severe DNA damage caused by the combination of olaparib and palbociclib. **Figure S3.** Colony formation assays of SUM149 cells treated with PARPi and/or CDK4/6i. **Figure S4.** The combined use of olaparib and palbociclib can inhibit the WNT pathway, which is not downregulated by single-agent olaparib treatment. **Figure S5.** Ser675 phosphorylation of β-catenin in the Wnt pathway mediates resistance to olaparib but can be inhibited by palbociclib. **Figure S6.** Growth curves of parental and acquired-resistant HCC1937 cells at different concentrations of olaparib. **Figure S7.** Weights of (A) MDA-MB-436 and (B) HCC1937 xenografted NOD-SCID mice.

## Data Availability

Please contact the corresponding author (Zhonghua Wang, wangzhonghua2691@sina.com) for data requests.

## Abbreviations

BC: Breast cancer
TNBC: Triple-negative breast cancer
ER: Oestrogen receptor
PR: Progesterone receptor
HER2: Human epidermal growth factor receptor 2
PARP: Poly-(ADP)-ribose polymerase
PARPi: PARP inhibitors
CDK: Cyclin-dependent kinase
CDK4/6i: CDK4/6 inhibitors
HR: Homologous recombination
DSBs: Double-stranded breaks
FDA: Food and Drug Administration
LAR: Luminal androgen receptor
CCK-8: Cell counting kit-8
CI: Combination index
CCD: Charge-coupled device
BSA: Bovine serum albumin
GFP: Green fluorescent protein
TA: Triamcinolone acetonide
RIN: RNA integrity number
GSEA: Gene set enrichment analysis
GEO: Gene Expression Omnibus
PCR: Polymerase chain reaction
qRT-PCR: Quantitative reverse transcription PCR
WB: Western blot
BCA: Bicinchoninic acid
IP: Immunoprecipitation
shRNA: Short hairpin RNA
PEI: Polyethyleneimine
OE: Overexpression
KD: Knockdown
KO: Knockout
IHC: Immunohistochemistry
oHSVs: Oncolytic herpes simplex viruses
