# Supplementary material for: Efficacy and mechanism of the combination of PARP and CDK4/6 inhibitors in the treatment of triple-negative breast cancer
Source: J Exp Clin Cancer Res. 2021 Apr 8;40:122. doi: 10.1186/s13046-021-01930-w (PMC8028839; doi:10.1186/s13046-021-01930-w)
Supplement: Supplementary file 2 — Additional file 2: Figure S1. Synergistic response to combined PARPi and CDK4/6i treatment in BRCAmut/TNBCs. Figure S2. Severe DNA damage caused by the combination of olaparib and palbociclib. Figure S3. Colony formation assays of SUM149 cells treated with PARPi and/or CDK4/6i. Figure S4. The combined use of olaparib and palbociclib can inhibit the WNT pathway, which is not downregulated by single-agent olaparib treatment. Figure S5. Ser675 phosphorylation of β-catenin in the Wnt pathway mediates resistance to olaparib but can be inhibited by palbociclib. Figure S6. Growth curves of parental and acquired-resistant HCC1937 cells at different concentrations of olaparib. Figure S7. Weights of (A) MDA-MB-436 and (B) HCC1937 xenografted NOD-SCID mice. [file 13046_2021_1930_MOESM2_ESM.pdf]

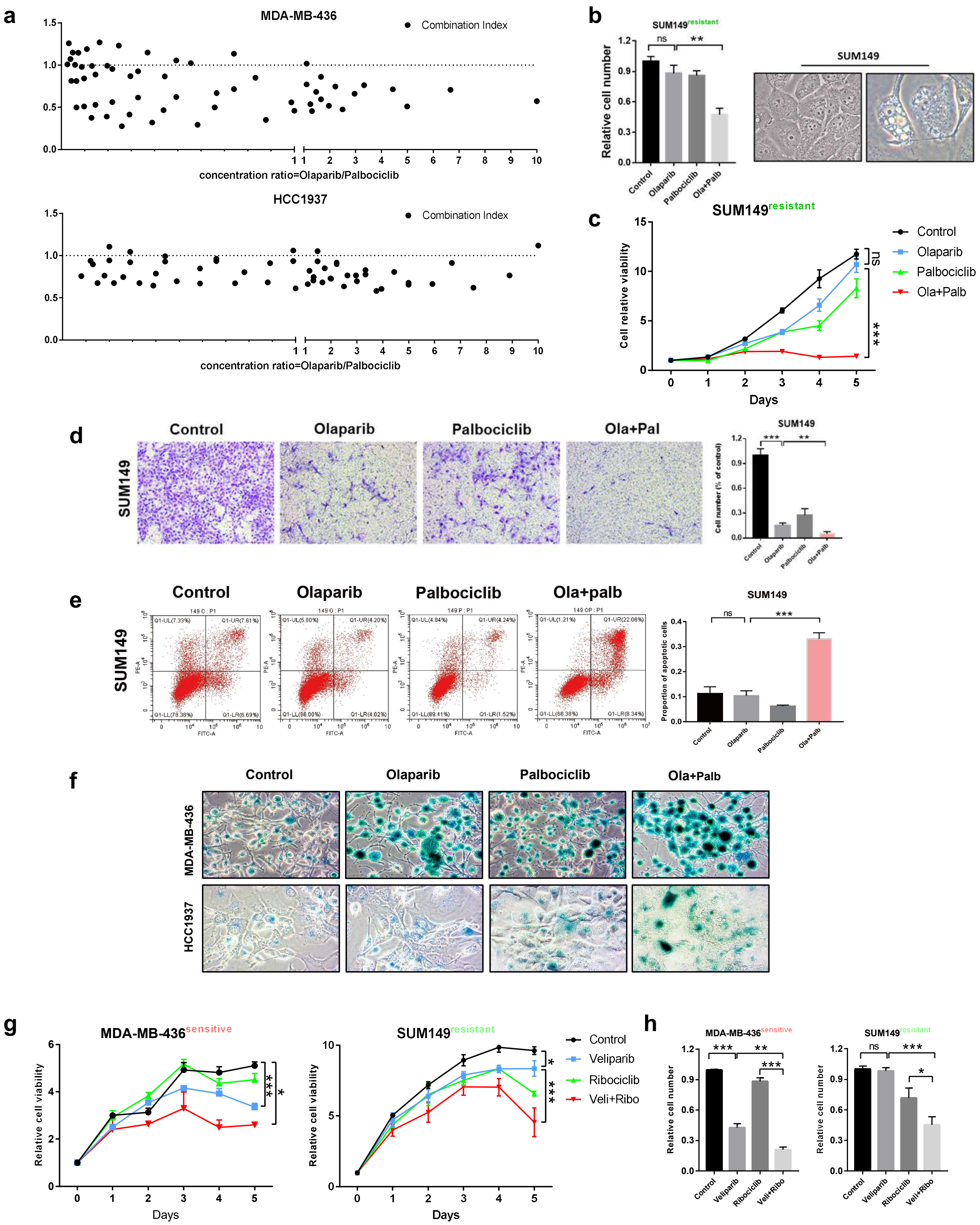

a
MDA-MB-436
Combination Index
concentration ratio=Olaparib/Palbociclib
HCC1937
Combination Index
concentration ratio=Olaparib/Palbociclib
b
SUM149resistant
Relative cell number
ns
**
Control
Olaparib
Palbociclib
Ola+Palb
SUM149
c
SUM149resistant
Cell relative viability
Days
Control
Olaparib
Palbociclib
Ola+Palb
ns
***
d
Control
Olaparib
Palbociclib
Ola+Pal
SUM149
Cell number (% of control)
e
Control
Olaparib
Palbociclib
Ola+palb
SUM149
FITC-A
PE-A
Proportion of apoptotic cells
f
Control
Olaparib
Palbociclib
Ola+Palb
MDA-MB-436
HCC1937
g
MDA-MB-436sensitive
Relative cell viability
Days
SUM149resistant
Control
Veliparib
Ribociclib
Veli+Ribo
h
MDA-MB-436sensitive
SUM149resistant
Relative cell number
Control
Veliparib
Ribociclib
Veli+Ribo

**Fig. S1 Synergistic response to combined PARPi and CDK4/6i treatment in BRCA$^{mut}$/TNBCs.**

**a** The combination index (CI) of (*Up*) MDA-MB-436 and (*Down*) HCC1937 cells treated with varying concentrations of olaparib and palbociclib for 5 days. The CI was calculated by the Chou-Talalay method with CompuSyn software.

**b** Changes in relative cell numbers and cell morphology after three days of single-agent or combined treatment in in PARPi-resistant cells SUM149.

**c** Cell proliferation curve of olaparib or palbociclib alone or in combination in SUM149 cells.

**d** Transwell migration assay in SUM149 cells pretreated with drug as indicated for 3 days. The migration times of SUM149 was 24 h.

**e** Apoptosis evaluation after three days of single-agent or combined treatment in SUM149 cells. c(olaparib)=15 μM, c(palbociclib)=15 μM.

**f** Cell senescence of (*Up*) MDA-MB-436 and (*Down*) HCC1937 cells was determined by the β-Galactosidase Staining Kit after treatment with 5 μM olaparib or 5 μM palbociclib alone or in combination.

**g** Cell proliferation curve of 5 μM veliparib or 5 μM ribociclib alone or combined in (*Left*) olaparib-sensitive MDA-MB-436 and (*Right*) olaparib-resistant SUM149 cells.

**h** Changes in the relative cell numbers after three days of single-agent or combined treatment.

Student's t-test; ***p < 0.001, **p < 0.01, *p < 0.05; ns, not significant. The data are presented as the means ± SEMs. Con: Control; Ola: olaparib; Palb: palbociclib; Veli: Veliparib; Ribo: Ribociclib.

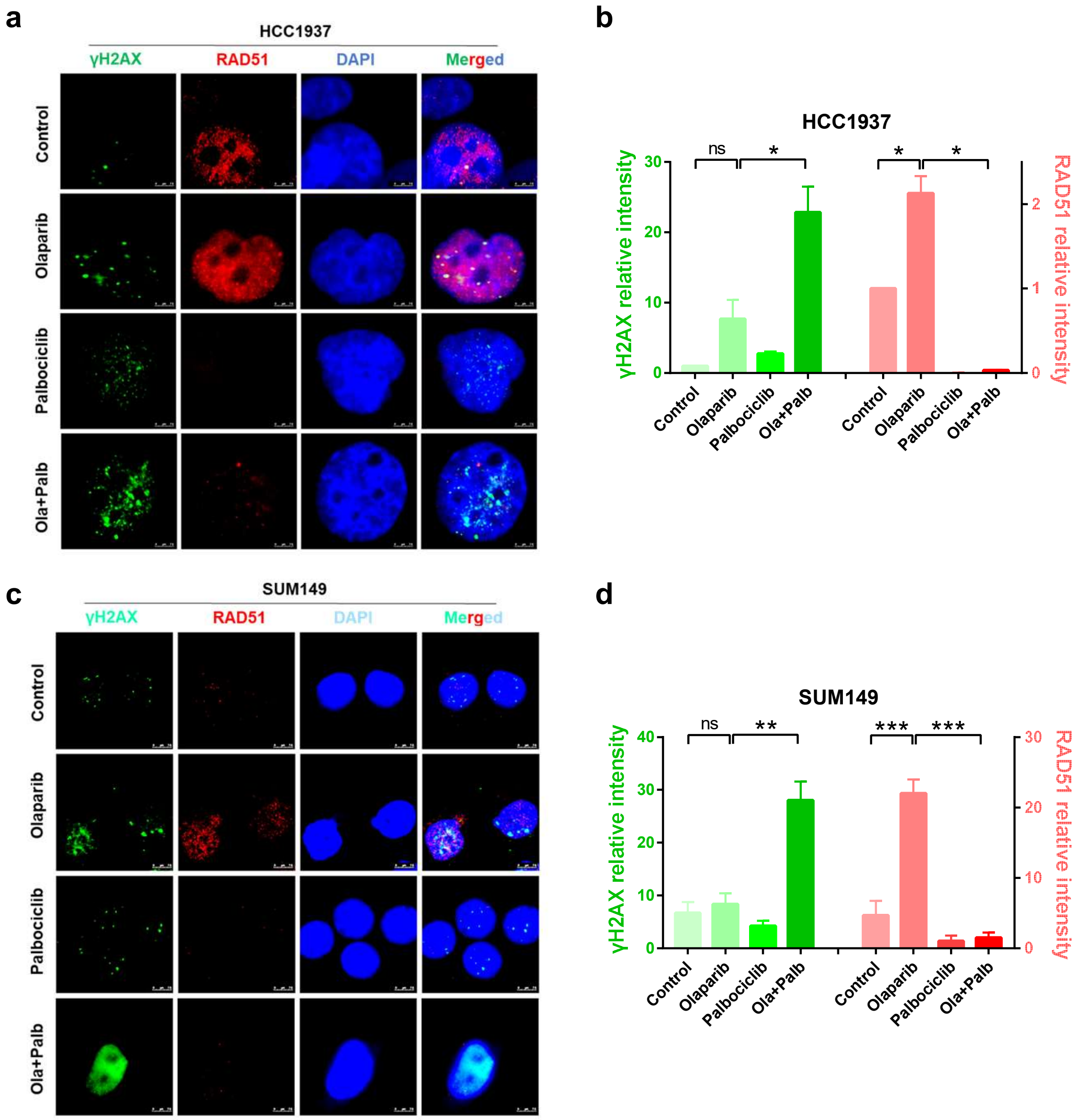


**Fig. S2 Severe DNA damage caused by the combination of olaparib and palbociclib.**
**a** Representative images of immunofluorescent staining of DAPI, γH2AX and RAD51 in HCC1937 cells treated with vehicle, 5 μM olaparib, 5 μM palbociclib or their combination for 72 h.
**b** The quantification of γH2AX and RAD51 in HCC1937 signal intensity was evaluated by ImageJ.
**c** Representative images of immunofluorescent staining of DAPI, γH2AX and RAD51 in SUM149 cells treated with as Figure S2a.
**d** The quantification of γH2AX and RAD51 in SUM149 signal intensity was evaluated by ImageJ. Scale bar, 7.5 μm.

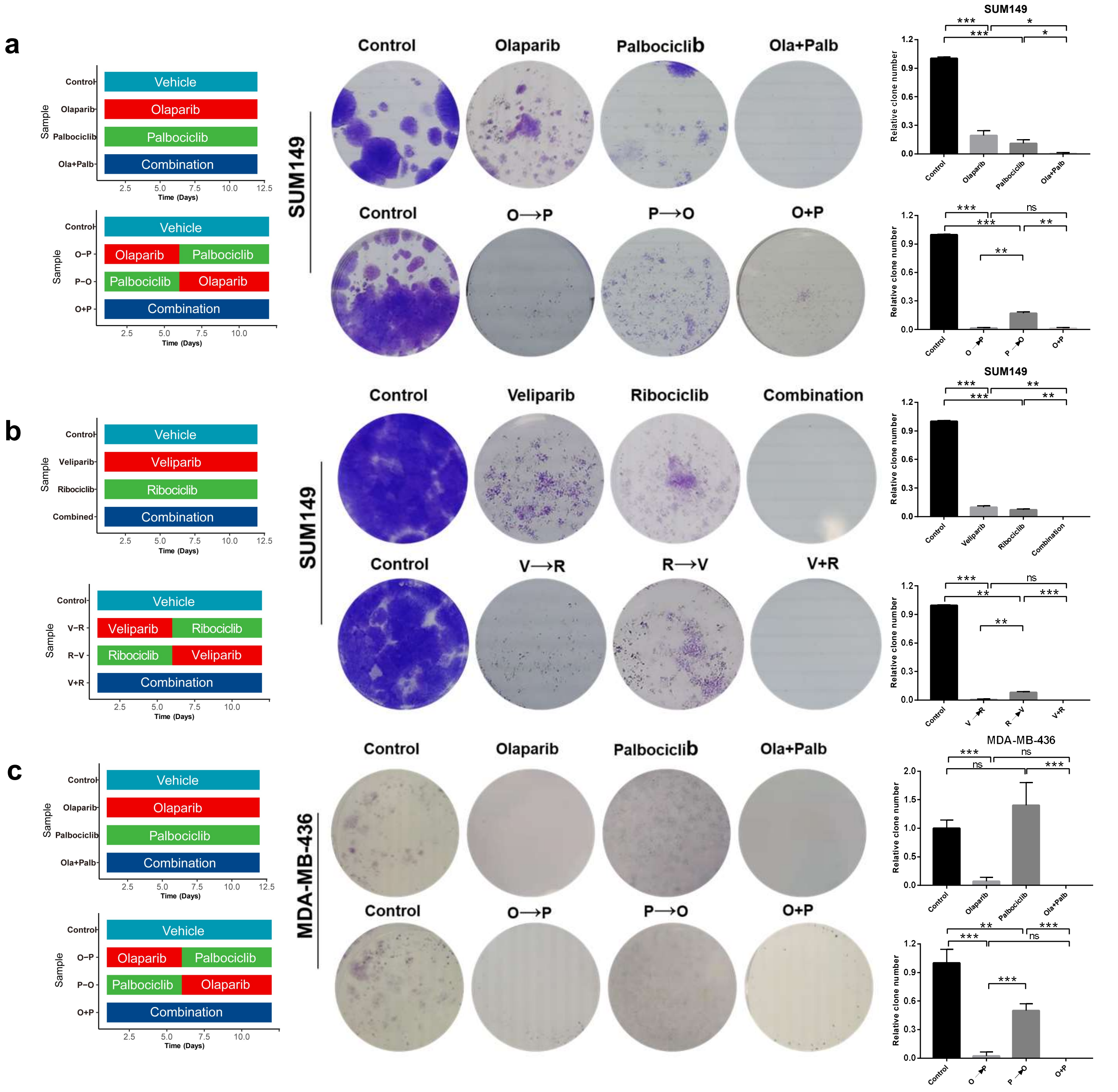


**Fig. S3. Colony formation assays of MDA-MB-436 and SUM149 cells treated with PARPi and/or CDK4/6i.**

**a** Colony formation assays of SUM149 cell lines treated with vehicle, 0.5 μM olaparib, 0.5 μM palbociclib or the combination for 12 days. The effect of the 6-day olaparib→6-day palbociclib sequential application (O→P) and its reverse (P→O) effect were also tested.

**b** Colony formation assays of SUM149 cell lines treated with vehicle, 0.5 μM veliparib, 0.5 μM ribociclib or the combination for 12 days. The effect of the 6-day veliparib →6-day ribociclib sequential application (V→R) and its reverse (R→V) effect were also tested. (*Left*) Pattern diagram of the sequential administration. (*Middle*) Representative images of colony formation assays. (*Right*) Quantification of colony formation.

**c** Colony formation assays of MDA-MB-436 cell lines treated as Fig. S3a.

Student's t-test; ***p < 0.001, **p < 0.01, *p < 0.05; ns, not significant. The data are presented as the means ± SEMs. Con: Control; Ola/O: Olaparib; Palb/P: Palbociclib; Veli/V: Veliparib; Ribo/R: Ribociclib.

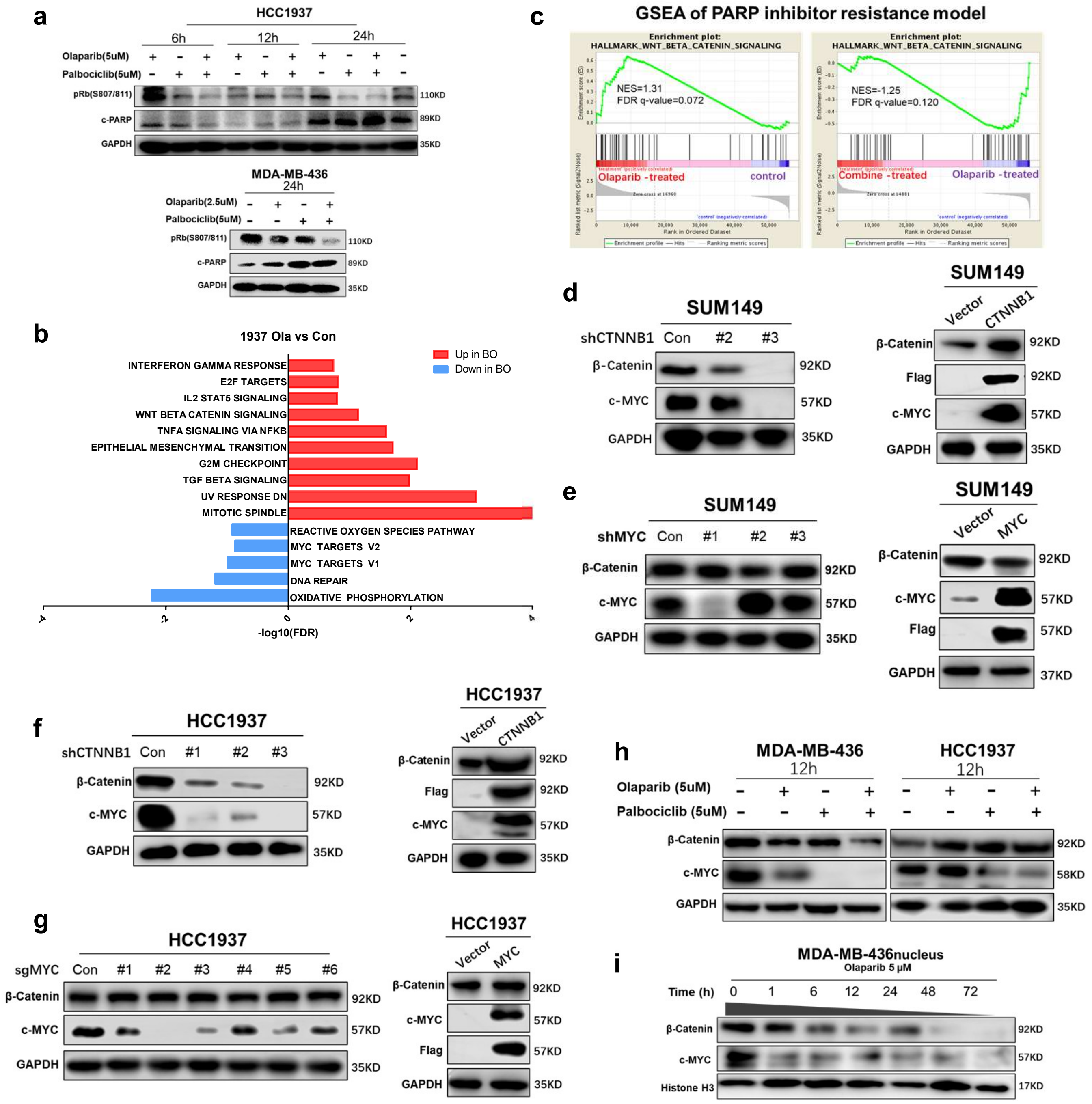
a
HCC1937
6h
12h
24h
Olaparib(5uM)
Palbociclib(5uM)
pRb(S807/811)
c-PARP
GAPDH
110KD
89KD
35KD
MDA-MB-436
24h
Olaparib(2.5uM)
Palbociclib(5uM)
b
1937 Ola vs Con
Up in BO
Down in BO
INTERFERON GAMMA RESPONSE
E2F TARGETS
IL2 STAT5 SIGNALING
WNT BETA CATENIN SIGNALING
TNFA SIGNALING VIA NFKB
EPITHELIAL MESENCHYMAL TRANSITION
G2M CHECKPOINT
TGF BETA SIGNALING
UV RESPONSE DN
MITOTIC SPINDLE
REACTIVE OXYGEN SPECIES PATHWAY
MYC TARGETS V2
MYC TARGETS V1
DNA REPAIR
OXIDATIVE PHOSPHORYLATION
-log10(FDR)
c
GSEA of PARP inhibitor resistance model
Enrichment plot: HALLMARK_WNT_BETA_CATENIN_SIGNALING
NES=1.31
FDR q-value=0.072
Olaparib -treated
control
NES=-1.25
FDR q-value=0.120
Combine -treated
Olaparib -treated
d
SUM149
shCTNNB1 Con #2 #3
β-Catenin
c-MYC
GAPDH
92KD
57KD
35KD
Vector
CTNNB1
Flag
e
shMYC Con #1 #2 #3
MYC
37KD
f
HCC1937
shCTNNB1 Con #1 #2 #3
g
sgMYC Con #1 #2 #3 #4 #5 #6
h
MDA-MB-436
HCC1937
12h
Olaparib (5uM)
Palbociclib (5uM)
58KD
i
MDA-MB-436nucleus
Olaparib 5 μM
Time (h) 0 1 6 12 24 48 72
Histone H3
17KD

**Fig. S4 The combined use of olaparib and Palbociclib can inhibit the WNT pathway, which is not downregulated by single-agent olaparib treatment.**

**a** Western blot (WB) analysis showing the level of phospho-Rb (Ser807/811) and cleaved PARP (Asp214) over time with drugs as indicated.

**b** GSEA results in olaparib-treated olaparib-resistant HCC1937 cells (BO) compared to vehicle-treated cells (BC).

**c** The Hallmark WNT_beta_catenin_signaling gene sets analyzed by GSEA which were upregulated in olaparib-treated HCC1937 cells (*Left)* but downregulated in combined-treated HCC1937 cells (*Right)*.

**d** WB analysis showing the levels of c-myc in CTNNB1-knockdown (CTNNB1 KD) or overexpression (CTNNB1 OE) SUM149 cells.

**e** WB analysis showing levels of β-catenin in MYC-knockout (MYC KO) or overexpression (MYC OE) SUM149 cells.

**f** WB analysis showed the levels of c-myc in CTNNB1-knockdown (CTNNB1 KD) or CTNNB1-overexpressing (CTNNB1 OE) HCC1937 cells.

**g** WB analysis showed the levels of β-catenin in MYC-knockout (MYC KO) or MYC-overexpressing (MYC OE) HCC1937 cells.

**h** WB analysis showing total levels of β-catenin and c-myc in MDA-MB-436 and HCC1937 cells treated with drugs as indicated for 12 h.

**i** Changes in nuclear β-catenin and c-myc protein levels in MDA-MB-436 over time under the indicated treatment.

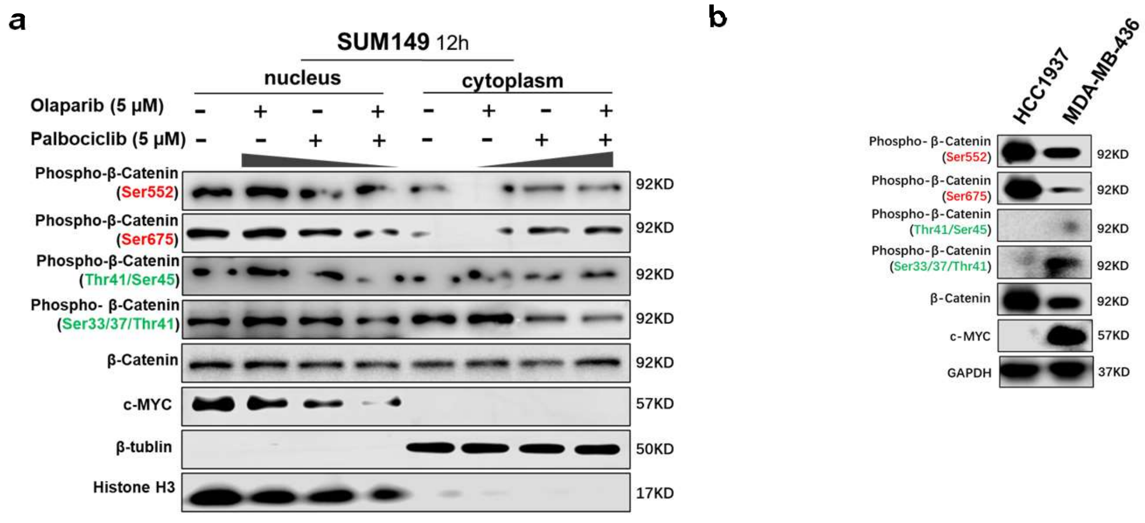


**Fig. S5 Ser675 phosphorylation of β-catenin in the Wnt pathway mediates resistance to olaparib but can be inhibited by palbociclib.**

**a** Western blot (WB) analysis showing the phosphorylation level of specific sites of β-catenin and c-myc in SUM149 cells treated with drugs as indicated for 12 h.

**b** WB analysis showing the levels of β-catenin, phosphorylated β-catenin (pβ-cateninSer552, pβ-cateninSer675, pβ-cateninThr41/Ser45 and pβ-cateninSer33/37/Thr41) and c-myc in the primary olaparib-sensitive MDA-MB-436 cells and olaparib-resistant HCC1937 cells. The phosphorylation of the sites marked in green negatively regulates the Wnt signaling pathway, while the sites marked in red promote its nuclear translocation, thereby positively regulating the Wnt signaling pathway.

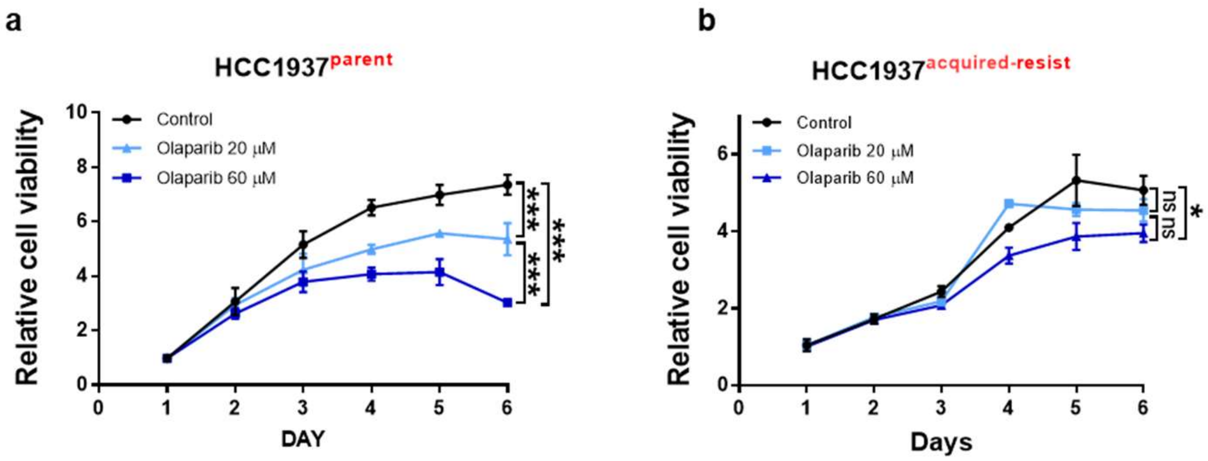


**Fig. S6 Growth curves of parental and acquired-resistant HCC1937 cells at different concentrations of olaparib.** CCK-8 analysis of parental HCC1937 cells (**a**) and acquired-resistant cells (**b**) under the treatment of 20 μM or 60 μM olaparib.
Student's t-test; ***$p < 0.001$, **$p < 0.01$, *$p < 0.05$; ns, not significant. The data are presented as the means ± SEMs

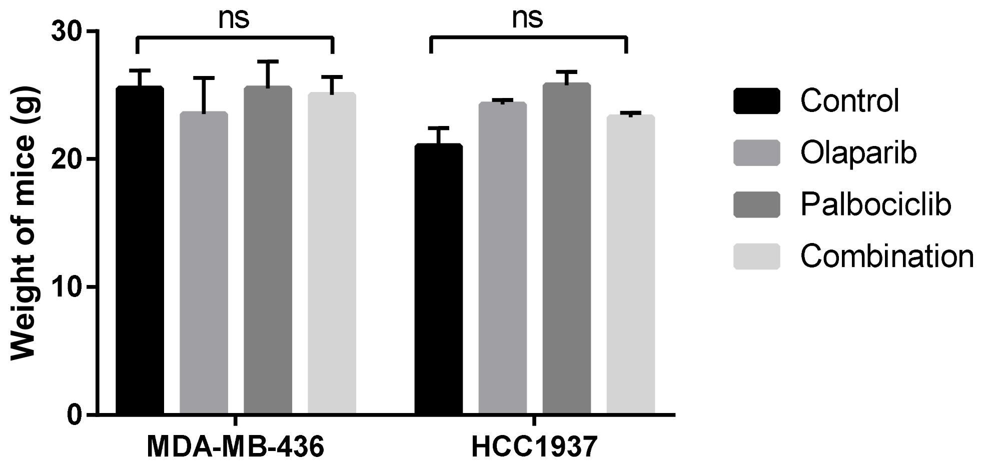


**Fig. S7 Weight of (A) MDA-MB-436 and (B) HCC1937 xenografted NOD-SCID mice.** Mice were treated with olaparib (50 mg/kg/day) and palbociclib (100 mg/kg/day), either alone or in combination, for 21 days.

One-way ANOVA; ns, not significant. The data are presented as the means ± SEMs.
